# Mapping phenotypic and genetic relationships among irritability, depression and ADHD in adolescence using network analysis

**DOI:** 10.1111/jcpp.70040

**Published:** 2025-09-20

**Authors:** Amy Shakeshaft, Luis C. Farhat, Charlotte A. Dennison, Olga Eyre, Olakunle Oginni, Michael C. O'Donovan, Argyris Stringaris, Ellen Leibenluft, Guilherme V. Polanczyk, Lucy Riglin, Anita Thapar

**Affiliations:** ^1^ Division of Psychological Medicine and Clinical Neurosciences, Wolfson Centre for Young People's Mental Health Cardiff University Cardiff UK; ^2^ Division of Psychological Medicine and Clinical Neurosciences, Centre for Neuropsychiatric Genetics and Genomics Cardiff University Cardiff UK; ^3^ Department of Psychiatry, Faculdade de Medicina FMUSP Universidade de Sao Paulo Sao Paulo Brazil; ^4^ Faculty of Brain Sciences, Division of Psychiatry University College London London UK; ^5^ Division of Psychology and Language Sciences University College London London UK; ^6^ Office of the Scientific Director National Institute of Mental Health Bethesda MD USA

**Keywords:** Irritability, ADHD, depression, oppositional defiant disorder, comorbidity, genetics, ALSPAC

## Abstract

**Background:**

Irritability is a common reason for referral to child and adolescent mental health services. However, debate exists as to whether irritability is best conceptualised and treated as a feature of mood disorder, oppositional defiant disorder or a core symptom of ADHD.

**Methods:**

We use network analyses to examine the relationships between adolescent irritability, headstrong/hurtful ODD items, depression and ADHD phenotypes, and polygenic scores (PGS) for depression and ADHD using the Avon Longitudinal Study of Parents and Children (ALSPAC). In primary analysis, irritability, depression, headstrong/hurtful ODD items and ADHD were defined using the Development and Well‐Being Assessment (DAWBA) at age 15. In secondary analysis, phenotypes were defined using the Short Mood and Feelings Questionnaire (SMFQ) and the Strengths and Difficulties Questionnaire (SDQ) ADHD and behavioural subscales at age 13. Finally, we tested for network replicability using confirmatory network analysis in the Millennium Cohort Study (MCS).

**Results:**

Results of network analyses using the DAWBA in ALSPAC indicated irritability was most strongly associated with headstrong/hurtful ODD items, followed by ADHD and depression. When including PGS, we observed an edge between irritability and depression PGS but not between irritability and ADHD PGS. Irritability appeared to be the primary pathway between ADHD and depression as well as between headstrong/hurtful ODD items and depression. Results were similar using SMFQ/SDQ in ALSPAC and confirmatory network analysis indicated excellent model fit in MCS.

**Conclusions:**

Although irritability appears to be transdiagnostic, phenotypically, it was most strongly associated with headstrong/hurtful ODD items and broader behavioural problems, which favours the ICD‐11 approach of including irritability as a specifier of ODD. However, irritability appeared to be a key connector between both ADHD and behavioural problems to depression; thus, is important to monitor and treat in affected youth with ADHD or behavioural problems.

## Introduction

Irritability, defined as a proneness to anger that may reach an impairing extent, is strongly associated with negative outcomes and is a common reason for referral to child and adolescent mental health services (Mikita & Stringaris, 2013). It is a feature of major depressive disorder (MDD), behavioural disorders (e.g. conduct/oppositional disorders) and neurodevelopmental conditions (e.g. ADHD) in young people (Leibenluft et al., 2024). There are currently multiple ways of conceptualising irritability, which have important implications for clinical management: (a) a mood problem (disruptive mood dysregulation disorder, DMDD), as in DSM‐5 (American Psychiatric Association, 2013); (b) an oppositional defiant disorder (ODD) specifier, as in ICD‐11 (World Health Organization, 2019); or (c) a core feature of ADHD (Karalunas, Gustafsson, Fair, Musser, & Nigg, 2019).

One approach used to conceptualise psychopathologies is by examining genetic validators, for example, investigating whether irritability shows genetic overlap with mood, behavioural or neurodevelopmental conditions. An early twin study (Stringaris, Zavos, Leibenluft, Maughan, & Eley, 2012) showed that the irritable (rather than headstrong/hurtful) dimension of ODD predicted later depression and showed genetic correlation with depression. This has been replicated (Waldman, Rowe, Boylan, & Burke, 2021), adding support to the distinction between irritability as a possible mood‐based construct compared to behavioural (headstrong/hurtful) ODD symptoms. Further support for this distinction comes from findings that, while nonshared environmental influences make a substantial contribution to irritability, unlike behavioural problems, shared environmental factors do not (Burt, 2009; Hettema et al., 2020; Stringaris et al., 2012). However, not all research is consistent, with some studies suggesting that irritability predicts and shows genetic overlap with ADHD (Merwood et al., 2014) and antisocial behaviour (Mikolajewski, Taylor, & Iacono, 2017).

Other genetic studies have used polygenic scores (PGS) to index common genetic liability for psychiatric disorders and tested associations with irritability. These have suggested associations between childhood irritability and ADHD PGS (Green, Baroud, Disalvo, Faraone, & Biederman, 2022; Nigg et al., 2020; Riglin et al., 2017) and depression PGS (Dissanayake et al., 2023; Riglin et al., 2019), with one study suggesting a specific association between depression PGS and adolescent‐onset irritability (Riglin et al., 2019). Therefore, evidence regarding the genetic aetiology of irritability is mixed, making it unclear whether irritability is better conceptualised as a mood, behavioural or neurodevelopmental condition. Further research is needed, particularly during adolescence when rates of mood problems sharply increase and associations between irritability and depression may, therefore, be clearer than in childhood.

In this study, we simultaneously examined the relationships among adolescent irritability, ADHD, depression and ODD phenotypes, as well as PGS, using network analyses in a large UK population cohort, the Avon Longitudinal Study of Parents and Children (ALSPAC). We further tested the consistency of the findings using different measures of irritability, ADHD, a broader measure of behavioural problems and depression, as well as in another cohort, the UK Millennium Cohort Study (MCS).

## Methods

### Sample

Our primary sample is ALSPAC, a longitudinal study which recruited pregnant women in the Avon region of South‐West England, due to give birth between 1 April 1991 and 31 December 1992 (Boyd et al., 2013; Fraser et al., 2013; Northstone et al., 2019). The core sample consisted of 14,541 mothers; of these pregnancies, 13,988 children were alive at 1 year. Following initial recruitment, an additional 913 children were recruited in three phases. Data were collected from families repeatedly and managed using REDCap (Harris et al., 2009). The study website contains details of all the data that are available through a fully searchable data dictionary and variable search tool (http://www.bristol.ac.uk/alspac/researchers/our‐data/). For families with multiple births, we include the oldest sibling.

We also used data from MCS (www.cls.ioe.ac.uk/mcs), a nationally representative UK longitudinal population‐based cohort of children born between 2000 and 2001. The sampling design ensured adequate representation across England, Wales, Scotland and Northern Ireland, as well as an enriched sampling of families from ethnic minority backgrounds and from deprived areas of the UK. The initial recruited sample consisted of 18,818 cohort members from 18,552 families. Additional eligible families were recruited in 2003–2004, to a total of 19,519 cohort members from 19,244 families. We used data collected in 2015, when cohort members were 14 years of age. DNA samples were collected from families when children were aged 14 years (from 9,259 cohort members). MCS survey data can be accessed by bona fide researchers through the UK Data Service (https://doi.org/10.5255/UKDA‐Series‐2000031).

### Network variables

#### Phenotype measures

In ALSPAC, we used data from when participants were approximately 15 years of age to enable replication across cohorts. Mothers and offspring independently completed the Development and Well‐Being Assessment (DAWBA, Goodman, Ford, Richards, Gatward, & Meltzer, 2000), a structured research diagnostic interview covering emotional, behavioural and hyperactivity disorders.

In accordance with previous research (Stringaris & Goodman, 2009), irritability during the past 6 months was defined by three items from the parent‐reported ODD section of the DAWBA (severe temper tantrums, touchy and easily annoyed and angry and resentful), rated on a 3‐point scale (0–2, indicating no more than others, a little more than others, a lot more than others) and summed to yield a total score (0–6). We also included the parent‐reported headstrong/hurtful items (argues, defies, annoys, blames others, spiteful) from the ODD section of the DAWBA (Burke et al., 2014; Stringaris & Goodman, 2009). As before, each item is rated on a 3‐point scale and summed to yield a total score ranging 0–10.

Parent‐reports of the DAWBA were used to assess ADHD, and adolescent self‐reports were used to assess depression due to data availability. Scores for each disorder were aggregated into computer‐generated DAWBA band ratings indicating the probability of each of these disorders being present as follows: 0 (<0.1%), 1 (~0.5%), 2 (~3%), 3 (~15%), 4 (~50%), 5 (>70%) (Goodman, Heiervang, Collishaw, & Goodman, 2011). These bands have been well‐validated in community samples of British children, showing excellent agreement with clinician‐rated diagnoses (Goodman et al., 2011).

To enable replication in a second cohort, MCS, which only included the Strengths and Difficulties Questionnaire (SDQ, Goodman, 1997) and Short Mood and Feelings Questionnaire (SMFQ, Angold, Costello, Messer, & Pickles, 1995), we repeated analyses using those measures at age 13 in ALSPAC and age 14 in MCS, due to data availability. Irritability was characterised using the item ‘Often has temper tantrums’ from the behavioural problems subscale of the parent‐reported SDQ. This item has previously been used to investigate irritability in young people in large population‐based studies (Henriksen et al., 2021) and is correlated with the sum of three irritability items in the DAWBA in multiple studies when both measures (SDQ and DAWBA) were parent‐reported (ALSPAC: *r* = .39, *p* < .001; Krebs et al., 2013: *r* = .49, *p* < .001). The SDQ hyperactivity/ADHD subscale (range 0–10) and a modified version of the SDQ behavioural problems subscale (without the irritability item (Srinivasan et al., 2023), range 0–8) were used to characterise ADHD and SDQ‐defined behavioural problems. Both subscales have been shown to identify ADHD and ODD, respectively, in adolescents (Vugteveen, De Bildt, Theunissen, Reijneveld, & Timmerman, 2021). Depression symptoms were obtained from the self‐reported SMFQ (range 0–26).

#### Genetics

We used PGS to index common genetic liability for ADHD and depression based on summary statistics from the following GWAS: (a) ADHD – Demontis et al. (2023) and (b) Depression – Adams et al. (2025). For full details of PGS calculation see Supporting Information. We were unable to generate PGS for ODD since, to our knowledge, there are no adequately powered GWAS. Each PGS was residualised against ancestry‐specific principal components and standardised to aid interpretation.

### Statistical analysis

All analyses were conducted in R (R Core Team, 2021). For a summary of all network analyses undertaken, see Table S1.

#### Network estimation

We first calculated cross‐sectional partial correlation networks in ALSPAC. In the first model (‘Phenotype only’), we used the DAWBA phenotypes exclusively. In the second model (‘Phenotype + PGS’), we incorporated PGS. In network models, each node represents a phenotype measure or a PGS, while each weighted edge connecting the nodes represents the correlation between two variables after conditioning on all other variables in the network. Missing data for any two given variables were excluded in a pairwise manner. Sample sizes for each pairwise comparison is presented in Table S2.

Networks were generated using the function ‘ggmModSelect’ in the R package, *bootnet* (Epskamp, Borsboom, & Fried, 2018), where input partial correlations were from Spearman's rank due to ordinal data. This method of network estimation searches for an optimal Gaussian graphical model (GGM) by minimising the Extended Bayesian Information Criterion (EBIC) of unregularised GGM models and has been shown to perform optimally in large samples, compared to other network estimation techniques (Isvoranu & Epskamp, 2023). The algorithm estimates 100 regularised network models with a range of sparsity levels (i.e., with the fewest vs. the greatest number of nodes and edges) using gLASSO (graphical least absolute shrinkage and selection operator). For each of the 100 networks, a specific set of edges is obtained. Models are then re‐fitted without regularisation to compute edge weights. The optimal model is chosen based on minimising EBIC, using the average sample size of pairwise correlations. Finally, individual edges of the selected network model are adjusted to improve fit through stepwise estimation.

Networks were plotted using the Fruchterman‐Reingold algorithm in *qgraph* (Epskamp, Cramer, Waldorp, Schmittmann, & Borsboom, 2012). The accuracy of network parameters was investigated using *bootnet* (Epskamp et al., 2018), where 1,000 nonparametric bootstraps were calculated for all network edge weights. We used the ‘differenceTest’ function in *bootnet*, which examines bootstrapped confidence intervals for edge weights to determine whether two edge weights can be considered significantly different.

For secondary analysis, we repeated network estimation using SDQ/SMFQ phenotypes and PGS in ALSPAC, using the same methods. Finally, confirmatory network estimation was conducted in MCS using two models proposed in Piazza et al. (2024), using *psychonetrics* R package. In the first confirmatory model, we extracted the network structures derived in ALSPAC (i.e., an adjacency matrix indicating zero and nonzero edges), then assessed how well these structures fit in MCS using standard fit indices (Root Mean Square Error of Approximation, RMSEA; Comparative Fit Index, CFI; Relative Fit Index, RFI). In the second confirmatory model, we assessed whether edge weights were comparable across cohorts by evaluating the fit of the model with equality constraints on network edges across cohorts, i.e., a model in which all ALSPAC and MCS edges were set to be equal. When testing the replication of ‘Phenotype + PGS’ networks in MCS we included only PGS from individuals of European ancestry, since the ALSPAC cohort is mostly of European ancestry and genetic liability cannot not reliably be characterised in non‐European ancestries using PGS derived from European ancestry GWAS studies (Dennison et al., 2024). For information about how genetic ancestry was derived see (Shakeshaft et al., 2024).

### Sensitivity analyses

Since ADHD and depression PGS are highly correlated, two additional ‘Phenotype + PGS’ networks containing only ADHD PGS, and only Depression PGS were estimated (see Supporting Information).

To check for the influence of missing data on network estimation, we re‐estimated networks excluding missing data in a listwise manner (i.e. estimated networks in complete cases only). We also performed multiple imputation using the *mice* R package (Van Buuren & Groothuis‐Oudshoorn, 2011), where 56 imputed datasets were generated for individuals with complete genetic data (*n* = 8,591) using predictive mean matching with 20 iterations. Missingness rates for phenotype variables in those with genetic data are presented in Table S3, with a maximum missingness rate of 56%. All variables in the networks were included in the imputation models, along with prior measurements of DAWBA phenotypes at age 13 as auxiliary variables. To test for the influence of missing data on the presence of edges, we estimated networks in each imputed dataset and confirmed the percentage of times each edge was present. To test for the influence of missing data on edge weights, we used the ‘micombine.cor’ function from *miceadds* (Robitzsch & Grund, 2024) to calculate a combined Spearman's correlation matrix from all imputed datasets, which we then used as the input to estimate a single network from all imputed datasets, using the ‘modelsearch’ function in *psychonetrics* (Eskamp, 2023) (equivalent to ‘ggmModselect’ as used in main analysis).

To see whether the network structure changed depending on the definition of behavioural problems, we modelled networks in ALSPAC using the conduct disorder (rather than headstrong/hurtful ODD) DAWBA bands at age 15, generated as for ADHD and depression, with ratings indicating the probability of conduct disorder being present (detailed above), in place of the headstrong/hurtful ODD dimension. Using the same method as in the primary analysis, we tested how well this network structure replicated across measures (SDQ/SMFQ) in ALSPAC using standard fit indices (RMSEA, CFI, RFI).

We estimated DAWBA networks separately in males and females, as previous evidence indicates differing associations of male and female irritability (Vidal‐Ribas et al., 2023). We then compared networks using the ‘NCT’ function from the *NetworkComparisonTest* R package (Van Borkulo et al., 2023), performing a network invariance test, a global strength invariance test and an edge strength invariance test for all edges (using FDR corrections for multiple comparisons across edges). Female and male networks are presented in the Supporting Information, using the ‘averageLayout’ function in *qgraph* to compute the same, joint layout over both graphs.

To test for rater effects, since associations are typically stronger when using the same informant (Orchard, Pass, Cocks, Chessell, & Reynolds, 2019), we also estimated networks using parent‐reported SDQ‐defined behavioural problems, ADHD and irritability, and this time, we used parent‐reported SMFQ instead of self‐reported SMFQ.

Finally, since previous evidence indicates that associations of youth irritability with other phenotypes and PGS differ depending on developmental patterns of irritability (e.g. onset age and persistence) (Riglin et al., 2019), we performed sensitivity checks, including binary definitions of irritability based on onset age and persistence into adolescence. We estimated three additional networks: (a) first, using a binary definition of DAWBA at age 15 to check that networks did not substantially change when using binary vs. continuous irritability scores; (b) using a new variable of child‐persistent irritability, defined as irritability both in childhood and adolescence (ages 8 and 15) and (c) using adolescent‐onset irritability, defined as irritability only in adolescence (age 15) and not in childhood (age 8). For these networks, a binary definition of irritability was used based on scoring ≥1 on any one of the DAWBA irritability items. For full details of network estimation using these binary measures see Supporting Information.

## Results

### Primary network estimation using DAWBA variables in ALSPAC

**Figure 1:**
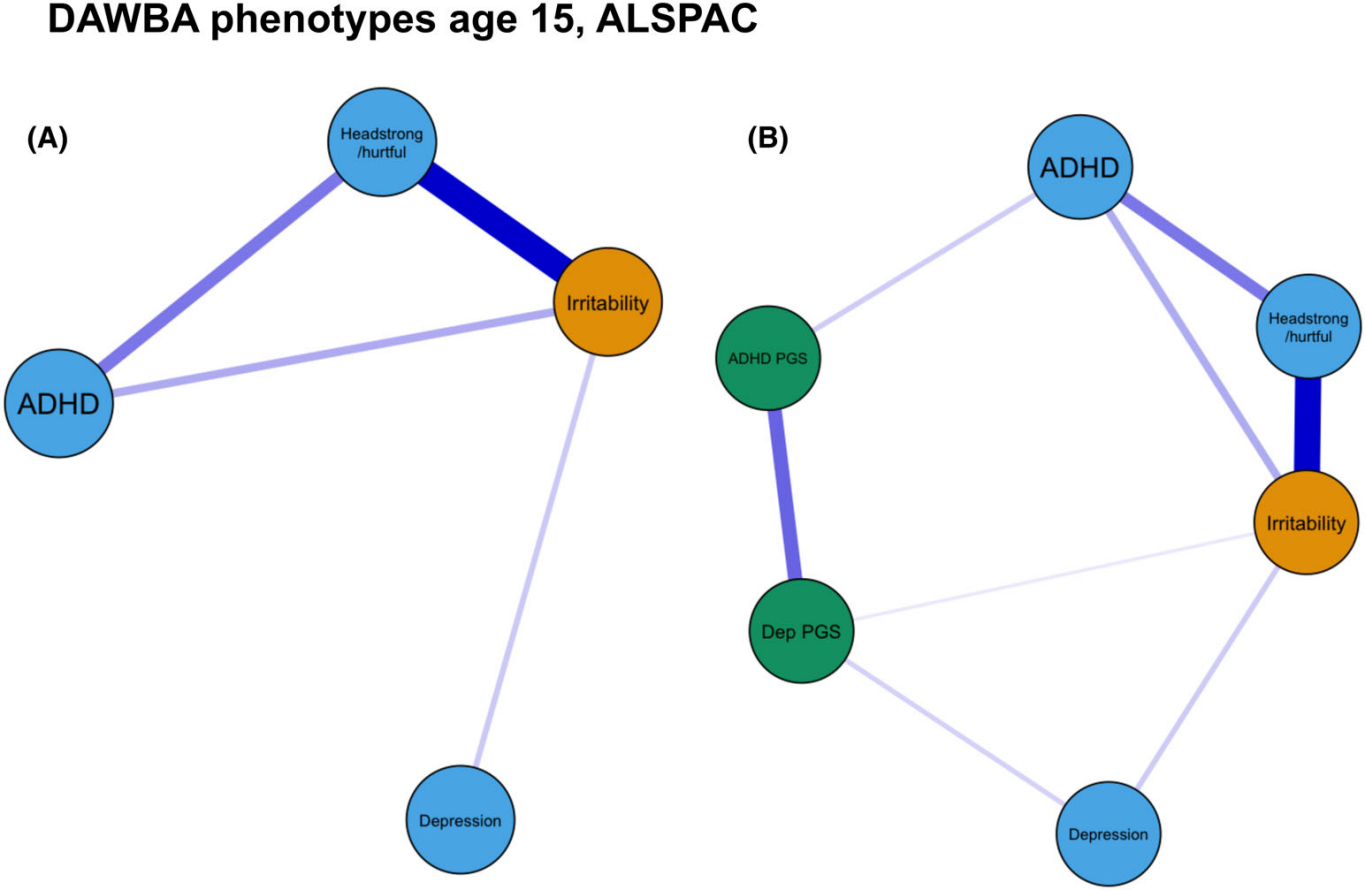
(A) ‘Phenotype only’ network using DAWBA variables at age 15 in ALSPAC. (B) ‘Phenotype + PGS’ network using DAWBA variables at age 15 in ALSPAC. In both networks edges represent Spearman's partial correlations between nodes. For absolute edge weights see Tables S4 and S5. Irritability, ADHD and headstrong/hurtful items are parent‐reported; depression is self‐reported

**Figure 2:**
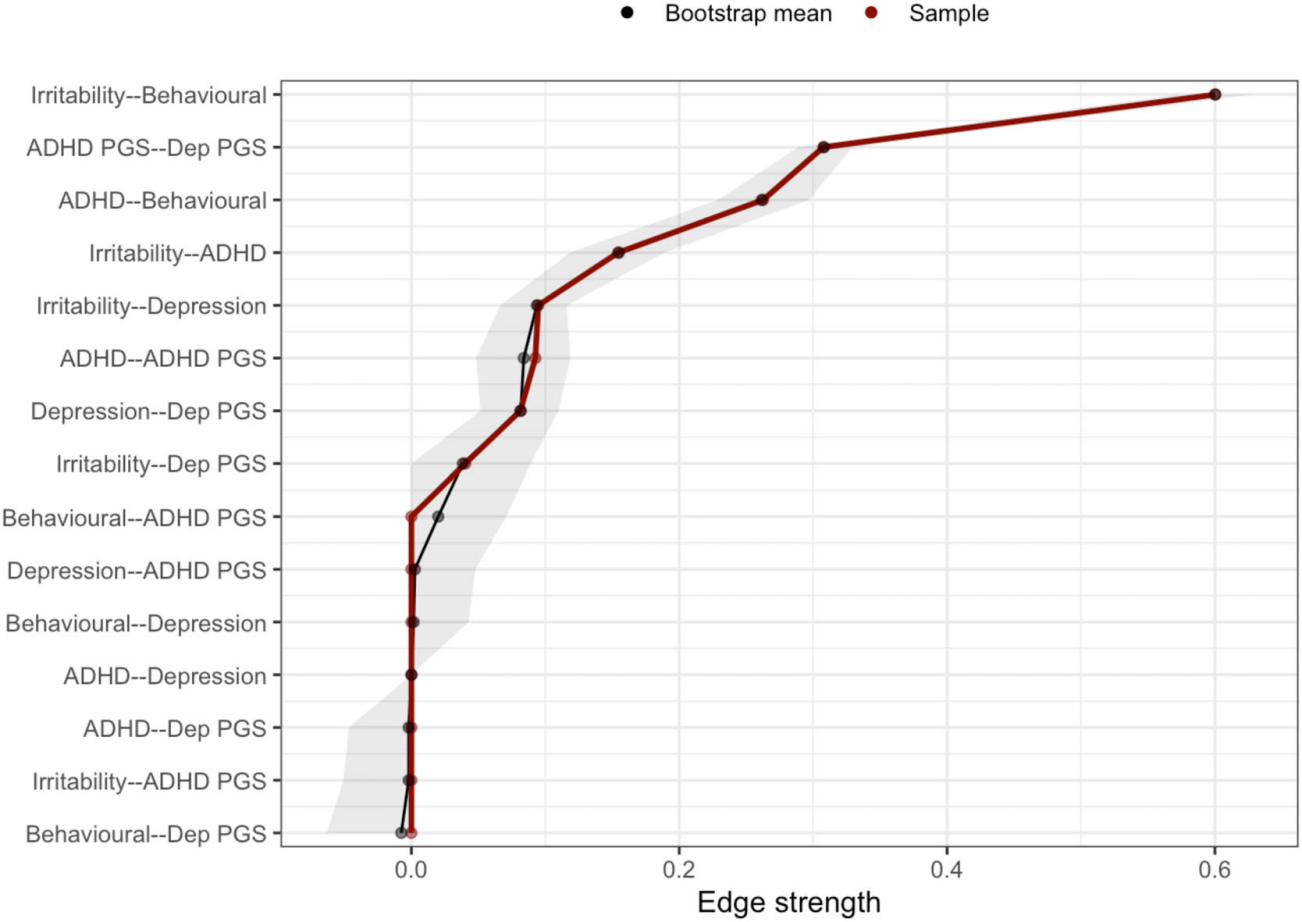
Comparison of ‘Phenotype + PGS’ network bootstrapped edge weights to sample network

In the ‘Phenotype only’ network (Figure 1A), irritability showed positive associations with all other phenotypes. Bootstrapped edge weights indicated that the strongest association existed between parent‐reported irritability and the headstrong/hurtful dimension of ODD, then between irritability and parent‐reported ADHD and finally, self‐reported depression (Figure 2). Depression did not show direct associations with either ADHD or headstrong/hurtful symptoms. In the ‘Phenotype + PGS’ network (Figure 1B) the same associations were present between phenotypes. Additionally, there were associations between depression PGS and depression and irritability phenotypes and between ADHD PGS and ADHD phenotype. Although an edge between irritability and ADHD PGS was not found in the network, there was limited evidence that the association between irritability and depression PGS was stronger than between irritability and ADHD PGS using bootstrapped edge weight confidence intervals (Figure S1).

The nonparametric bootstraps of the ‘Phenotype + PGS’ network showed edges were estimated accurately, as sample values were comparable to bootstrap mean edge weights (Figure 2).

### Secondary network estimation in ALSPAC using SDQ/SMFQ variables

**Figure 3:**
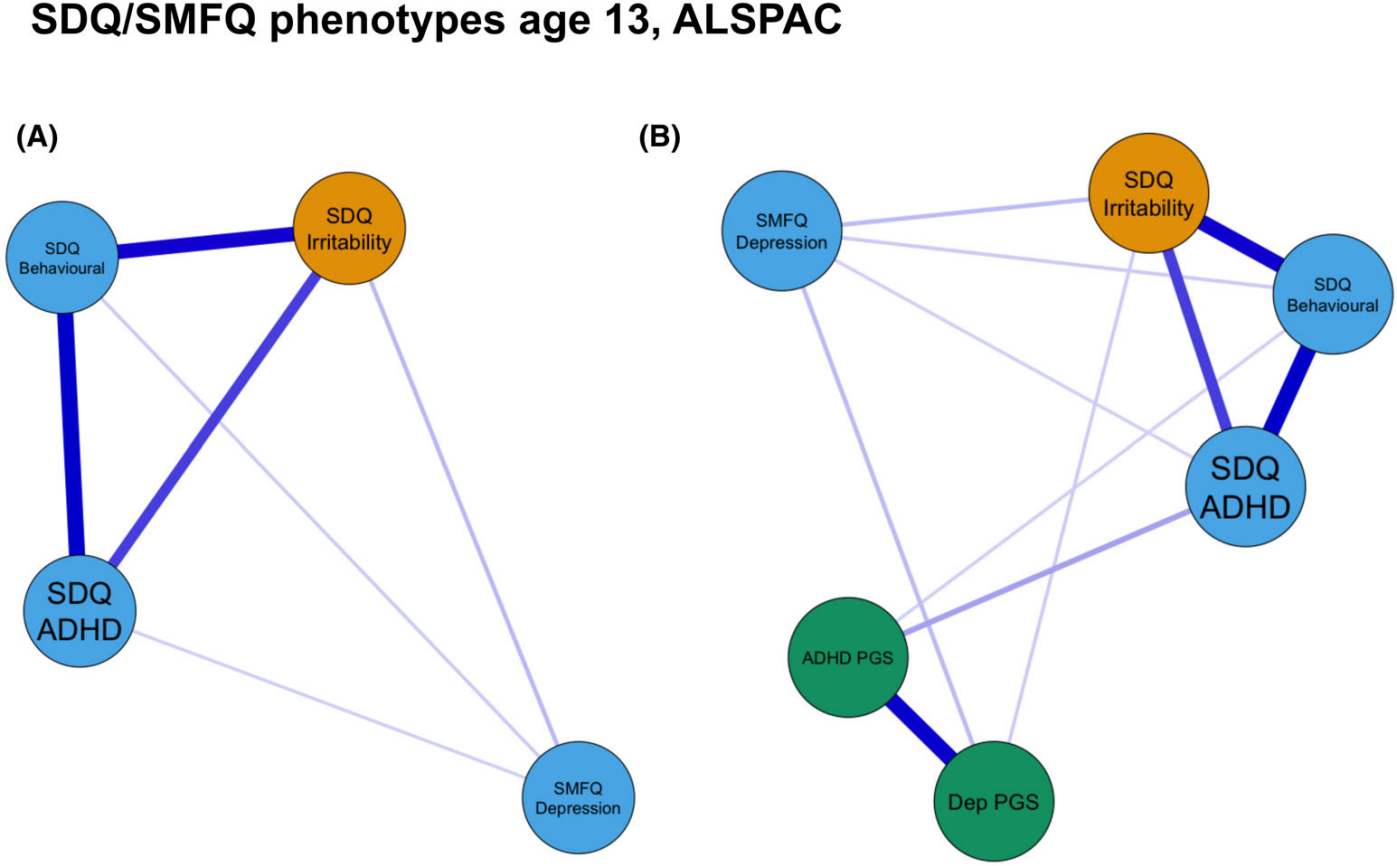
(A) ‘Phenotype only’ network SDQ/SMFQ variables at age 13 in ALSPAC. (B) ‘Phenotype + PGS’ network SDQ/SMFQ variables at age 13 in ALSPAC. In both networks edges represent Spearman's partial correlations between nodes. For absolute edge weights see Tables S6 and S7. Irritability is parent‐reported using the irritability item from parent‐rated SDQ; ADHD and behavioural problems are parent‐reported SDQ subscales; and depression is self‐reported SMFQ

For both ‘Phenotype only’ (Figure 3A) and ‘Phenotype + PGS’ (Figure 3B) networks, the same edges were present as in the primary networks, using DAWBA, with the addition of more broadly defined (questionnaire) depression showing direct associations with SDQ‐defined behavioural problems and ADHD. The structure of the DAWBA ‘phenotype only’ network showed good model fit when tested across SDQ/SMFQ phenotype variables, based on standard fit index thresholds (RMSEA = 0.05 [0.04, 0.07]; CFI = 0.99; RFI = 0.96). Constraining edges to be equal across measures resulted in poorer model fit (RMSEA = 0.15 [0.14, 0.16]; CFI = 0.88; RFI = 0.82), indicating the strength of associations between different phenotypes was not the same across measures.

### Confirmatory network estimation in MCS

Confirmatory network estimation of the SDQ/SMFQ ‘Phenotype + PGS’ ALSPAC network in a second dataset, MCS, showed excellent model fit for the presence of edges for the ‘Phenotype + PGS’ network (RMSEA = 0.01 [0.00, 0.02]; CFI = 1.0; RFI = 1.0), as well as edge weights for both the ‘Phenotype only’ (RMSEA = 0.02 [0.01, 0.03]; CFI = 1.0; RFI = 1.0) and ‘Phenotype + PGS’ networks (RMSEA = 0.01 [0.01, 0.02]; CFI =1.0; RFI = 1.0), thereby indicating this network was successfully replicated across cohorts. Model fit could not be tested for the presence of edges for the ‘Phenotype only’ network due to zero degrees of freedom.

### Sensitivity analysis

‘Phenotype + PGS’ networks containing only ADHD PGS and only Depression PGS (Figures S2A,B) showed a similar pattern of associations as the primary analysis, with only Depression PGS, not ADHD PGS, showing a direct association with irritability.

Networks estimated using complete cases (listwise deletion) showed the same edges present for the ‘Phenotype only’ network using DAWBA phenotypes in ALSPAC (*n* = 4,529, see Figure S3A) and the same edges present for the ‘Phenotype + PGS’ network (*n* = 3,732, Figure S3B) as in the primary analysis. Estimating networks on imputed datasets, to test for the consistency of edge presence, showed consistent edges, as presented in Table S8. Edge strengths in the network estimated using pooled Spearman's correlation coefficients from imputed datasets all fell within the 95% confidence intervals from of bootstrapped estimates of edge strengths from sampled data (Figure S4).

When examining the DAWBA conduct disorder subscale rather than headstrong/hurtful ODD items (Figure S5), unlike what was observed for headstrong/hurtful ODD items, the strongest edge was between irritability and ADHD. While the ‘Phenotype + PGS’ network showed an edge between depression PGS and conduct disorder and not between depression PGS and irritability, comparison of the strength of these associations suggested they were similar (Figure S6). This network structure showed good model fit when tested across SDQ/SMFQ phenotype variables (RMSEA = 0.03 [0.02, 0.03]; CFI = 0.99; RFI = 0.97). Constraining edges to be equal across measures also resulted in good model fit (RMSEA = 0.03 [0.03, 0.03]; CFI = 0.98; RFI = 0.96), indicating associations between phenotypic items were similar across the DAWBA and SDQ/SMFQ, when including the DAWBA conduct subscale.

Visual inspection of networks estimated separately in males and females (Figure S7) showed some differences in network structure and edges, including the edge between irritability and depression only being present in males. Instead, there was an edge between headstrong/hurtful ODD items and depression present in females. Additionally, the edge between depression PGS and irritability was only present in females and not males. However, overall network invariance (M = 0.10, *p* = .12), global strength invariance (S = 0.02, *p* = .70) and edge strength invariance tests (Table S9) indicated little evidence for sex differences between networks, indicating that visual differences in networks likely resulted from differences in sample size and/or sampling variation.

Including parent‐reported SMFQ in the network (Figure S8) showed the same edges present as when self‐reported SMFQ was included (Figure 3B); however, the strength of edges between depression and irritability, ADHD and headstrong/hurtful symptoms became stronger.

Networks generated using a binary definition of irritability showed similar results as the primary analysis (Figure S9). When we compared networks estimated using child‐persistent vs. adolescent‐onset irritability, all phenotypic associations showed a similar pattern of results; however, there was no association between depression PGS and childhood‐persistent irritability—only with adolescent‐onset irritability (Figure S10).

## Discussion

In this study, we simultaneously examined associations between adolescent irritability, ADHD, headstrong/hurtful ODD items and depression phenotypes and PGS using a network approach, with the aim of identifying whether adolescent irritability can be better conceptualised as a mood, behavioural or neurodevelopmental condition. Overall, our results provide evidence for adolescent irritability truly being transdiagnostic since, across measures, raters and cohorts, irritability was consistently associated with ADHD, depression and headstrong/hurtful ODD or SDQ‐defined behavioural problems, after adjusting for the relationships with other disorders. However, adolescent irritability showed the strongest link with headstrong/hurtful ODD compared to ADHD and depression. Similar findings were not observed in sensitivity analyses using conduct disorder rather than headstrong/hurtful ODD. In terms of genetic validators, irritability was more consistently associated with depression PGS than ADHD PGS, when controlling for all other variables.

Several studies have indicated univariate associations between irritability and a broad spectrum of child psychopathologies, including ADHD, depression and ODD (Leibenluft et al., 2024), and this is reflected in the different conceptualisations of irritability (i.e. as an ODD specifier in ICD‐11 and as a mood disorder in DSM‐5). However, this is the first time, to our knowledge, that phenotypic and genetic associations have been investigated within one model utilising a network approach. Methods such as these are important in characterising irritability, since comorbidity is the norm in child psychopathology (Fisher, 2022) and observed comorbidities may be explained by a third condition. Indeed, in our DAWBA network models, we found no direct association between self‐reported depression and parent‐reported ADHD or headstrong/hurtful symptoms, suggesting that irritability may be the key link in the relationship between depression and ADHD and the headstrong/hurtful ODD dimension. This is consistent with previous work indicating that irritability is a key mediator between childhood neurodevelopmental difficulties and adolescent depression (Eyre et al., 2019). However, to our knowledge, this has not previously been demonstrated for headstrong/hurtful ODD problems and depression. Future studies should also investigate where bipolar disorder, in which irritability is also a common feature, fits into this network; this was not feasible in the current work due to the low prevalence of bipolar disorder in population cohorts.

This network approach is also advantageous when examining genetic liability, as it allows for control of not only phenotypic overlap but also genetic correlation between psychiatric disorders such as ADHD and depression (Powell, Martin, Thapar, Rice, & Anney, 2021). This is exemplified here by the consistent association between irritability and depression PGS compared to the apparent lack of direct association between irritability and ADHD PGS in all network models. However, there was not a significant difference between these edge strengths, reflecting less certainty around these associations. Previous work has indicated associations between ADHD PGS and irritability in childhood and adolescence (Green et al., 2022; Nigg et al., 2020; Riglin et al., 2017, 2019). However, here, we see this association is not present when controlling for ADHD, depression and ODD headstrong/hurtful or broader behavioural problem phenotypes and depression PGS, which may be indicative that this association does not reflect direct genetic effects of ADHD risk alleles on irritability; rather, it is a consequence of ADHD itself and/or ODD headstrong/hurtful or broader behavioural problems being associated with greater irritability. This suggests that whilst irritability is a common feature of ADHD, it may not be a core aspect.

The relationship between adolescent irritability and depression PGS is consistent with previous research which suggests that depression PGS may be specifically associated with irritability emerging in adolescence, rather than in childhood (Riglin et al., 2019).

Currently, DSM‐5 (American Psychiatric Association, 2013) and ICD‐11 (World Health Organization, 2019) diagnostic criteria have differing approaches to conceptualising youth irritability. In DSM‐5, irritability primarily comes under DMDD, a mood disorder, whereby the clinical presentation of severe irritability without headstrong/hurtful symptoms would result in a DMDD diagnosis. In addition, in DSM‐5, irritability is also noted as a dimension of ODD, as a symptom of intermittent explosive disorder and as being common in other mood disorders and ADHD. However, in ICD‐11, irritability is primarily regarded as a specifier of ODD. When considering where irritability best sits, our results do not provide a clear‐cut answer. On one hand, they favour the ICD‐11 approach in that phenotypically, adolescent irritability is most strongly related to headstrong/hurtful ODD items and broader behavioural problems. However, at the genetic level, it shows the strongest relationship with depression liability. While this may favour the DSM‐5 approach, a caveat here is that it is uncertain whether this would still be the case if headstrong/hurtful ODD PGS were available. Furthermore, since psychiatric PGS show wide‐ranging associations with a wide range of psychiatric, neurodevelopmental and physical health phenotypes, the association between MDD PGS and irritability does not necessarily indicate irritability as a mood symptom. What is clear is that irritability appears to be an important symptom linking different types of youth psychopathology (mood, behavioural and neurodevelopmental conditions). Therefore, we suggest that diagnostic systems should acknowledge that irritability is likely to be present in each of these disorder categories and is an important specifier.

This work also highlights the important issue of rater effects when studying child psychopathology. We see that although the patterns of associations are consistent across raters, the strength of associations between irritability, ADHD, depression and headstrong/hurtful or broader behavioural problem phenotypes depends on whether symptoms are self‐ or parent‐reported. Specifically, the association between irritability and depression is stronger than the association between irritability and ADHD when all phenotypes are parent‐rated; however, when depression is self‐rated, the association between ADHD and irritability is stronger. Due to no available self‐report data for ADHD, headstrong/hurtful ODD items, SDQ‐defined behavioural problems, or irritability, we were unable to do a full exploration nor provide a complete explanation for these rater effects. However, this is an important consideration for future research, as previous work has shown discrepancies in these phenotypes depending on raters (Mallidi et al., 2023; Orchard et al., 2019; Zik et al., 2022).

Our results indicate that the network structure (i.e. presence of edges/associations between variables) replicates well across measures (DAWBA and SDQ/SMFQ); however, the strength of the associations replicates less well. This is likely due to the measures of behavioural problems differing across the DAWBA and SDQ. In the DAWBA, we use items indexing the headstrong/hurtful dimensions of ODD, whereas in the SDQ, we use the behavioural problems subscale, which includes items from the broader ICD‐11 category of conduct problems (F91), encompassing ODD and other types of conduct symptoms, including lying and stealing. We see better replication across measures when using the DAWBA conduct disorder diagnostic bands.

The present study has several important strengths. The good model fit of networks across different measures of irritability, ADHD, depression and headstrong/hurtful ODD and broader behavioural problems, as well as across two different cohorts indicates the network structure and associations shown are likely to be reliable, which is essential when performing network analyses that are inherently exploratory (Borsboom, Robinaugh, Rhemtulla, & Cramer, 2018). While the two population cohorts used in this study, ALSPAC and MCS, are both UK cohorts, they are demographically different. Firstly, there is a decade between these two birth cohorts, during which rates of depression have sharply increased (Armitage et al., 2023). Secondly, unlike ALSPAC, MCS contains more typically underrepresented populations, e.g. those from lower socio‐economic classes and higher proportions of ethnic minority populations. Therefore, the consistency of our findings across cohorts is even more notable and likely increases the generalisability of results. In addition, by using both the DAWBA and SDQ/SMFQ data in ALSPAC, we were able to investigate both clinical diagnoses of ADHD, depression diagnoses, as well as symptoms.

Our results should be viewed considering methodological limitations. Firstly, due to the lack of a well‐powered GWAS of ODD or conduct disorder, we were unable to explore the relationship between genetic liability to conduct/oppositional phenotypes and irritability, only genetic liability to ADHD and depression. This is an important avenue for future research since previous work suggest that irritability shows genetic overlap with antisocial behaviour (Mikolajewski et al., 2017). Further, the use of the one‐item (‘Often has temper tantrums’) irritability measure from the SDQ, which was used in our secondary analysis, is not ideal. However, this SDQ item has been used to investigate irritability in young people in other large population‐based studies (Henriksen et al., 2021) and this item demonstrated moderate correlation with the sum of the three parent‐reported DAWBA irritability items (Krebs et al., 2013). A further limitation when assessing the association between psychiatric PGS and irritability is the fact that we were unable to examine these associations in individuals of non‐European genetic ancestry. ALSPAC is predominantly of European genetic ancestry and while a strength of the MCS cohort is its ethnic diversity and sampling strategy, there are still too few individuals from non‐European genetic ancestries to be able to conduct well‐powered analyses in these populations. Additionally, as outlined above, we were unable to undertake a complete analysis of rater effects due to only parent‐reported irritability, ADHD and behavioural problems data available at the age of interest. Finally, using this method alone we are unable to determine any direction of association or causal relationships between variables in the network model. Other methods should be used to determine causality in the relationships between irritability, ADHD, depression and headstrong/hurtful ODD and broader behavioural problems, including longitudinal data, twin studies and Mendelian randomisation. This may have use for determining the role of specific interventions on child psychopathology.

## Conclusion

This study suggests that adolescent irritability appears to be genuinely transdiagnostic, showing robust associations with ADHD, depression and headstrong/hurtful ODD and broader behavioural problems across measures, raters and cohorts. However, the strong link with ODD headstrong/hurtful items and SDQ‐defined behavioural problems likely favours the ICD‐11 approach, which includes irritability as a specifier of ODD. Irritability appears to be a key link between ADHD and depression, as well as behavioural problems and depression. Thus, it is important to assess and monitor in youth alongside ADHD and/or behavioural problems.

## Ethical considerations

Ethical approval for the study was obtained from the ALSPAC Ethics and Law Committee and Local Research Ethics Committees. Informed consent for the use of data collected via questionnaires and clinics was obtained from participants following the recommendations of the ALSPAC Ethics and Law Committee. Ethical approval for MCS was gained from the Multi‐Centre Research Ethics Committee. Parents gave written informed consent. Consent for biological samples has been collected in accordance with the Human Tissue Act (2004).

**Key points**

**What's known?**

Irritability is a core feature of several child psychopathologies, including behavioural and mood disorders, as well as a common feature of neurodevelopmental conditions such as ADHD. However, there is mixed evidence on how it is best conceptualised when considering phenotypic and genetic associations.

**What's new?**

Network analysis showed the strongest association between youth irritability with ODD headstrong/hurtful items, then with ADHD and depression phenotypes, as well as with depression genetic liability.

**What's relevant?**

Irritability was the primary pathway between ADHD and depression phenotypes, as well as between behavioural problems and depression phenotypes; therefore, it should be monitored in youth with ADHD and/or behavioural problems.

## Supporting information


**Table S1.** Summary of network analyses.
**Table S2.** Pairwise sample sizes used for network calculation in ALSPAC at age 15.
**Table S3.** Number and % of missing data for variables included in the multiple imputation.
**Table S4.** Edge weights of ‘Phenotype only’ DAWBA network in ALSPAC.
**Table S5.** Edge weights of ‘Phenotype + PGS’ DAWBA network in ALSPAC.
**Table S6.** Edge weights ‘Phenotype only’ SDQ/SMFQ network in ALSPAC.
**Table S7.** Edge weights ‘Phenotype + PGS’ SDQ/SMFQ network in ALSPAC.
**Table S8.** Frequency (percentage of time) an edge was present in the imputed datasets (total *n* = 56).
**Table S9.** Results from edge strength invariance tests between ‘Phenotype + PGS’ DAWBA networks estimated separately in males and females.
**Figure S1.** Results of *differenceTest*, testing for differences in bootstrapped edge weights, as determined using the presence of overlapping confidence intervals.
**Figure S2.** (A) ‘Phenotype + PGS’ network containing only ADHD PGS. (B) ‘Phenotype + PGS’ network containing only Depression PGS.
**Figure S3.** (A) ‘Phenotype only’ network estimated with complete cases (*n* = 4,529). (B) ‘Phenotype + PGS’ network estimated with complete cases (*n* = 3,732).
**Figure S4.** Edge strengths (orange dots) of network estimated from imputed datasets, with range bars indicating 95% confidence intervals (CI) of sample network edge strength from bootstraps.
**Figure S5. (**A) ‘Phenotype only’ network using DAWBA variables at age 15 in ALSPAC, including a node for conduct disorder instead of headstrong/hurtful ODD symptoms. (B) ‘Phenotype + PGS’ network using DAWBA variables at age 15 in ALSPAC. In both networks edges represent Spearman's partial correlations between nodes.
**Figure S6.** Results of *differenceTest* the network including conduct disorder instead of the headstrong/hurtful ODD dimension to capture behavioural problems.
**Figure S7.** ‘Phenotype + PGS’ network estimated in females (top) and males (bottom).
**Figure S8.** ‘Phenotype + PGS’ network using all parent‐reported SDQ/SMFQ phenotypes.
**Figure S9.** Phenotype + PGS network estimated using a binary definition of irritability based on DAWBA in ALSPAC.
**Figure S10.** Phenotype & PGS networks estimated using irritability definitions based on age of onset. (A) Child‐persistent irritability and (B) adolescent‐onset irritability networks.

## Data Availability

Access to data from ALSPAC can be requested via the study website (http://www.bristol.ac.uk/alspac/researchers/access/). Millennium Cohort Study data are available from the UK Data Service: https://www.ukdataservice.ac.uk/.
