# Supplementary material for: Mapping phenotypic and genetic relationships among irritability, depression and ADHD in adolescence using network analysis
Source: J Child Psychol Psychiatry. 2025 Sep 20;67(3):333–43. doi: 10.1111/jcpp.70040 (PMC12883585; doi:10.1111/jcpp.70040)
Supplement: Supplementary file 1 — Table S1. Summary of network analyses. Table S2. Pairwise sample sizes used for network calculation in ALSPAC at age 15. Table S3. Number and % of missing data for variables included in the multiple imputation. Table S4. Edge weights of ‘Phenotype only’ DAWBA network in ALSPAC. Table S5. Edge weights of ‘Phenotype + PGS’ DAWBA network in ALSPAC. Table S6. Edge weights ‘Phenotype only’ SDQ/SMFQ network in ALSPAC. Table S7. Edge weights ‘Phenotype + PGS’ SDQ/SMFQ network in ALSPAC. Table S8. Frequency (percentage of time) an edge was present in the imputed datasets (total n = 56). Table S9. Results from edge strength invariance tests between ‘Phenotype + PGS’ DAWBA networks estimated separately in males and females. Figure S1. Results of differenceTest, testing for differences in bootstrapped edge weights, as determined using the presence of overlapping confidence intervals. Figure S2. (A) ‘Phenotype + PGS’ network containing only ADHD PGS. (B) ‘Phenotype + PGS’ network containing only Depression PGS. Figure S3. (A) ‘Phenotype only’ network estimated with complete cases (n = 4,529). (B) ‘Phenotype + PGS’ network estimated with complete cases (n = 3,732). Figure S4. Edge strengths (orange dots) of network estimated from imputed datasets, with range bars indicating 95% confidence intervals (CI) of sample network edge strength from bootstraps. Figure S5. (A) ‘Phenotype only’ network using DAWBA variables at age 15 in ALSPAC, including a node for conduct disorder instead of headstrong/hurtful ODD symptoms. (B) ‘Phenotype + PGS’ network using DAWBA variables at age 15 in ALSPAC. In both networks edges represent Spearman's partial correlations between nodes. Figure S6. Results of differenceTest the network including conduct disorder instead of the headstrong/hurtful ODD dimension to capture behavioural problems. Figure S7. ‘Phenotype + PGS’ network estimated in females (top) and males (bottom). Figure S8. ‘Phenotype + PGS’ network using all parent‐reported SDQ/SMFQ phenotyp [file JCPP-67-333-s001.docx]

**Supplementary Material**

**Polygenic score calculation**

Allelic information was checked against the HRC reference panel and any SNPs with non-matching alleles were excluded. Palindromic or ambiguous (CT/AG) variants were also excluded. Only common SNPs (MAF>0.01) were kept. This resulted in a sample of 3,976,009 SNPs passing all the above QC.

Discovery summary statistics from the largest available published GWAS were used for depression (Adams et al., 2025) and ADHD (Demontis et al., 2023). The summary statistics were processed to perform QC filtering, align SNPs against the HRC reference panel and convert summary data to a standardised format, using an R pipeline (available at <https://github.com/CardiffMRCPathfinder/summaRygwasqc>). None of the GWAS used to generate summary statistics included MCS or ALSPAC cohort members.

Polygenic scores (PGS) were calculated for each discovery phenotype in PLINK using the PRS continuous shrinkage (CS) approach (Ge, Chen, Ni, Feng & Smoller, 2019). This is a polygenic scoring approach that uses all available HapMap-3 (International HapMap et al., 2010) SNPs and adjusts the per-SNP effect sizes relative to their GWAS association signals, taking into account LD, using pre-computed LD information provided with PRS-CS, based on the European ancestry subset of the 1000 genomes phase 3 reference sample. PGS were calculated by summing the number of alleles (weighted by the adjusted effect size) across the full set of SNPs for each person. The PRS-CS-auto approach was used, which automatically detects the sparseness of the genetic architecture for each discovery phenotype, based on the discovery summary statistics.

**Validation of SDQ irritability item in ALSPAC**

In our secondary analyses, irritability was characterised using the item “Often has temper tantrums” from the conduct disorder/behavioural problems subscale of the parent-reported SDQ. This item has previously been used to investigate irritability in young people in large population-based studies (Henriksen et al., 2021) and has previously demonstrated robust correlation (r=0.49, p<0.001) with the sum of three irritability items in the DAWBA (Goodman, Ford, Richards, Gatward & Meltzer, 2000; Stringaris & Goodman, 2009), when both measures (SDQ and DAWBA) were parent-reported (Krebs et al., 2013). In ALSPAC, the spearman’s rank correlation between the parent-reported SDQ irritability item and irritability measured using the DAWBA, both at age 13, was 0.36, p<2x10^-16^.

We also calculated the polygenic score (PGS) for self-reported irritability from a GWAS in UK Biobank (from MRC IEU OpenGWAS (ID: UKB-b-13745) (Elsworth et al., 2020), 125,001 cases; 317,168 controls; see <https://biobank.ctsu.ox.ac.uk/crystal/field.cgi?id=1940> for details of irritability measure), to calculate whether genetic liability to irritability was associated with the response to this item. PGS were calculated as detailed above. There was evidence for an association between irritability PGS and the parent SDQ irritability item (b=0.04 SE±0.009, p1.5x10^-5^), to a similar, degree to the association between irritability PGS and the DAWBA irritability items (b=0.06 ±0.014, p=1.7x10^-5^).

**Network estimation using binary irritability definitions**

Networks were generated using the function ‘mgm’ in the R package, *bootnet* (Epskamp, Borsboom & Fried, 2018). This method estimates a mixed graphical model, allowing input of both categorical and continuous variables, whereby irritability was binary, and the rest of the variables were continuous. Continuous variables were rank transformed due to being skewed, this is equivalent to using Spearman correlations (Isvoranu & Epskamp, 2023), as in the main analysis.

**Supplementary Tables**

**Table S1** – Summary of network analyses.

| **Analysis** | **Network type** | **Purpose** | **Cohort** | **Variables** | **Age** | **Informant** | **Subset of cohort?** | **Figure** |
| --- | --- | --- | --- | --- | --- | --- | --- | --- |
| Primary | Phenotype only | - | ALSPAC | **DAWBA**: Irritability, Depression, Headstrong/hurtful, ADHD | 15 | Irritability: parent, ADHD: parent, Headstrong/hurtful: parent, Depression: self | - | Figure 1A |
| Primary | Phenotype + PGS | - | ALSPAC | **DAWBA**: Irritability, Depression, Headstrong/hurtful, ADHD;  **ADHD PGS,**  **Depression PGS** | 15 | Irritability: parent, ADHD: parent, Headstrong/hurtful: parent, Depression: self | - | Figure 1B |
| Secondary | Phenotype only | Replication across measures | ALSPAC | **SDQ**: Irritability, Behavioural problems, ADHD;  **SMFQ**: Depression, | 13 | Irritability: parent, ADHD: parent, Behavioural problems: parent, Depression: self | - | Figure 3A |
| Secondary | Phenotype + PGS | Replication across measures | ALSPAC | **SDQ**: Irritability, Behavioural problems, ADHD;  **SMFQ**: Depression;  **ADHD PGS,**  **Depression PGS** | 13 | Irritability: parent, ADHD: parent, Behavioural problems: parent, Depression: self | - | Figure 3B |
| Secondary | Phenotype only | Replication across cohorts | MCS | **SDQ**: Irritability, Behavioural problems, ADHD;  **SMFQ**: Depression | 14 | Irritability: parent, ADHD: parent, Behavioural problems: parent, Depression: self | - | NA |
| Secondary | Phenotype + PGS | Replication across cohorts | MCS | **SDQ**: Irritability, Behavioural problems, ADHD;  **SMFQ**: Depression;  **ADHD PGS,**  **Depression PGS** | 14 | Irritability: parent, ADHD: parent, Behavioural problems: parent, Depression: self | - | NA |
| Sensitivity | Phenotype + PGS | Individual PGS associations | ALSPAC | **DAWBA**: Irritability, Depression, Headstrong/hurtful, ADHD;  **ADHD PGS** | 15 | Irritability: parent, ADHD: parent, Headstrong/hurtful: parent, Depression: self | - | Figure S2A |
| Sensitivity | Phenotype + PGS | Individual PGS associations | ALSPAC | **DAWBA**: Irritability, Depression, Headstrong/hurtful, ADHD;  **Depression PGS** | 15 | Irritability: parent, ADHD: parent, Headstrong/hurtful: parent, Depression: self | - | Figure S2B |
| Sensitivity | Phenotype only | Missing data check | ALSPAC | **DAWBA**: Irritability, Depression, Headstrong/hurtful, ADHD | 15 | Irritability: parent, ADHD: parent, Headstrong/hurtful: parent, Depression: self | Complete cases | Figure S3A |
| Sensitivity | Phenotype + PGS | Missing data check | ALSPAC | **DAWBA**: Irritability, Depression, Headstrong/hurtful, ADHD;  **ADHD PGS,**  **Depression PGS** | 15 | Irritability: parent, ADHD: parent, Headstrong/hurtful: parent, Depression: self | Complete cases | Figure S3B |
| Sensitivity | Phenotype | Conduct disorder | ALSPAC | **DAWBA**: Irritability, Depression, Conduct disorder, ADHD; | 15 | Irritability: parent, ADHD: parent, Conduct disorder: parent,  Depression: self | - | Figure S5A |
| Sensitivity | Phenotype + PGS | Conduct disorder | ALSPAC | **DAWBA**: Irritability, Depression, Conduct disorder, ADHD;  **ADHD PGS,**  **Depression PGS** | 15 | Irritability: parent, ADHD: parent, Conduct disorder: parent,  Depression: self | - | Figure S5B |
| Sensitivity | Phenotype + PGS | Sex differences | ALSPAC | **DAWBA**: Irritability, Depression, Headstrong/hurtful, ADHD;  **ADHD PGS,**  **Depression PGS** | 15 | Irritability: parent, ADHD: parent, Headstrong/hurtful: parent, Depression: self | Females | Figure S7 |
| Sensitivity | Phenotype + PGS | Sex differences | ALSPAC | **DAWBA**: Irritability, Depression, Headstrong/hurtful, ADHD;  **ADHD PGS,**  **Depression PGS** | 15 | Irritability: parent, ADHD: parent, Headstrong/hurtful: parent, Depression: self | Males | Figure S7 |
| Sensitivity | Phenotype + PGS | Rater differences | ALSPAC | **SDQ**: Irritability, Behavioural problems, ADHD;  **SMFQ**: Depression;  **ADHD PGS,**  **Depression PGS** | 13 | Irritability: parent, ADHD: parent, Behavioural problems: parent, Depression: parent | - | Figure S8 |
| Sensitivity | Phenotype + PGS | Binary definition of irritability | ALSPAC | **DAWBA**: Irritability (binary), Depression, Headstrong/hurtful, ADHD | 15 | Irritability: parent, ADHD: parent, Headstrong/hurtful: parent,  Depression: self | - | Figure S9 |
| Sensitivity | Phenotype + PGS | Irritability age at onset check | ALSPAC | **DAWBA**: Irritability (childhood persistent), Depression, Headstrong/hurtful, ADHD;  **ADHD PGS,**  **Depression PGS** | 8 & 15 | Irritability: parent, ADHD: parent, Headstrong/hurtful: parent,  Depression: self | - | Figure S10A |
| Sensitivity | Phenotype + PGS | Irritability age at onset check | ALSPAC | **DAWBA**: Irritability (adolescent-onset), Depression, Headstrong/hurtful, ADHD;  **ADHD PGS,**  **Depression PGS** | 8 & 15 | Irritability: parent, ADHD: parent, Headstrong/hurtful: parent,  Depression: self | - | Figure S10B |

**Table S2 –** Pairwise sample sizes used for network calculation in ALSPAC at age 15. Italic values on the diagonal indicate sample size for individual variable. Irritability = DAWBA items, parent rated; ADHD = DAWBA bands, parent-rated, Behavioural problems = DAWBA items, parent rated; Depression = DAWBA bands, self-rated; ADHD PGS = ADHD polygenic scores; Depression PGS = depression polygenic scores.

|  | **Irritability** | **ADHD** | **Behavioural problems** | **Depression** | **ADHD PGS** | **Depression PGS** |
| --- | --- | --- | --- | --- | --- | --- |
| **Irritability** | *4588* |  |  |  |  |  |
| **ADHD** | 4587 | *4693* |  |  |  |  |
| **Behavioural problems** | 4559 | 4572 | *4573* |  |  |  |
| **Depression** | 4559 | 4664 | 4544 | *5285* |  |  |
| **ADHD PGS** | 3775 | 3860 | 3767 | 4331 | *8591* |  |
| **Depression PGS** | 3775 | 3860 | 3767 | 4331 | 8591 | *8591* |

**Table S3 –** Number and % of missing data for variables included in the multiple imputation.

|  | **N missing** | **%** |
| --- | --- | --- |
| **Depression** | 4260 | 50 |
| **ADHD** | 4731 | 55 |
| **Irritability** | 4816 | 56 |
| **Behavioural problems** | 4824 | 56 |
| **ADHD PGS** | 0 | 0 |
| **Depression PGS** | 0 | 0 |

**Table S4 –** Edge weights of ‘Phenotype only’ DAWBA network in ALSPAC.

|  | **Irritability** | **ADHD** | **Behavioural problems** | **Depression** |
| --- | --- | --- | --- | --- |
| **Irritability** | 0 | 0.16 | 0.60 | 0.10 |
| **ADHD** | 0.16 | 0 | 0.26 | 0 |
| **Behavioural problems** | 0.60 | 0.26 | 0 | 0 |
| **Depression** | 0.10 | 0 | 0 | 0 |

**Table S5 –** Edge weights of ‘Phenotype + PGS’ DAWBA network in ALSPAC.

|  | **Irritability** | **ADHD** | **Behavioural problems** | **Depression** | **ADHD PGS** | **Depression PGS** |
| --- | --- | --- | --- | --- | --- | --- |
| **Irritability** | 0 | 0.15 | 0.23 | 0.12 | 0 | 0.04 |
| **ADHD** | 0.15 | 0 | 0.20 | 0 | 0.08 | 0 |
| **Behavioural problems** | 0.60 | 0.26 | 0 | 0 | 0.06 | 0 |
| **Depression** | 0.09 | 0 | 0 | 0 | 0 | 0.08 |
| **ADHD PGS** | 0 | 0.09 | 0.06 | 0 | 0 | 0.31 |
| **Depression PGS** | 0.04 | 0 | 0 | 0.08 | 0.31 | 0 |

**Table S6 –** Edge weights ‘Phenotype only’ SDQ/SMFQ network in ALSPAC.

|  | **Irritability** | **ADHD** | **Behavioural problems** | **Depression** |
| --- | --- | --- | --- | --- |
| **Irritability** | 0 | 0.21 | 0.29 | 0.07 |
| **ADHD** | 0.21 | 0 | 0.33 | 0.05 |
| **Behavioural problems** | 0.29 | 0.33 | 0 | 0.05 |
| **Depression** | 0.07 | 0.05 | 0.05 | 0 |

**Table S7 –** Edge weights ‘Phenotype + PGS’ SDQ/SMFQ network in ALSPAC.

|  | **Irritability** | **ADHD** | **Behavioural problems** | **Depression** | **ADHD PGS** | **Depression PGS** |
| --- | --- | --- | --- | --- | --- | --- |
| **Irritability** | 0 | 0.21 | 0.29 | 0.07 | 0 | 0.05 |
| **ADHD** | 0.21 | 0 | 0.32 | 0.05 | 0.10 | 0 |
| **Behavioural problems** | 0.29 | 0.32 | 0 | 0.05 | 0.05 | 0 |
| **Depression** | 0.07 | 0.05 | 0.05 | 0 | 0 | 0.06 |
| **ADHD PGS** | 0 | 0.10 | 0.05 | 0 | 0 | 0.31 |
| **Depression PGS** | 0.05 | 0 | 0 | 0.07 | 0.31 | 0 |

**Table S8 –** Frequency (percentage of time) an edge was present in the imputed datasets (total n=56).

|  | **Irritability** | **ADHD** | **Behavioural problems** | **Depression** | **ADHD PGS** | **Depression PGS** |
| --- | --- | --- | --- | --- | --- | --- |
| **Irritability** | 0.0 | 100.0 | 100.0 | 100.0 | 0.00 | 92.9 |
| **ADHD** | 100.0 | 0.0 | 100.0 | 3.6 | 100.0 | 0.0 |
| **Behavioural problems** | 100.0 | 100.0 | 0.0 | 3.6 | 8.9 | 0.0 |
| **Depression** | 100.0 | 3.6 | 3.6 | 0.0 | 16.1 | 100.0 |
| **ADHD PGS** | 0.0 | 100.0 | 8.9 | 16.1 | 0.0 | 100.0 |
| **Depression PGS** | 92.9 | 0.0 | 0.00 | 100.0 | 100.0 | 0.0 |

**Table S9 –** Results from edge strength invariance tests between ‘Phenotype + PGS’ DAWBA networks estimated separately in males and females. P-values are corrected for multiple comparisons using FDR.

| **Edge 1** | **Edge 2** | **Test statistic (E)** | **P-value** |
| --- | --- | --- | --- |
| Irritability | ADHD | 0.01 | 1.00 |
| Irritability | Behavioural problems | 0.06 | 0.35 |
| ADHD | Behavioural problems | 0.02 | 1.00 |
| Irritability | Depression | 0.09 | 0.07 |
| ADHD | Depression | 0.00 | 1.00 |
| Behavioural problems | Depression | 0.10 | 0.07 |
| Irritability | ADHD PGS | 0.00 | 1.00 |
| ADHD | ADHD PGS | 0.02 | 1.00 |
| Behavioural problems | ADHD PGS | 0.00 | 1.00 |
| Depression | ADHD PGS | 0.00 | 1.00 |
| Irritability | Depression PGS | 0.05 | 1.00 |
| ADHD | Depression PGS | 0.00 | 1.00 |
| Behavioural problems | Depression PGS | 0.00 | 1.00 |
| Depression | Depression PGS | 0.01 | 1.00 |
| ADHD PGS | Depression PGS | 0.03 | 0.52 |

**Supplementary Figures**


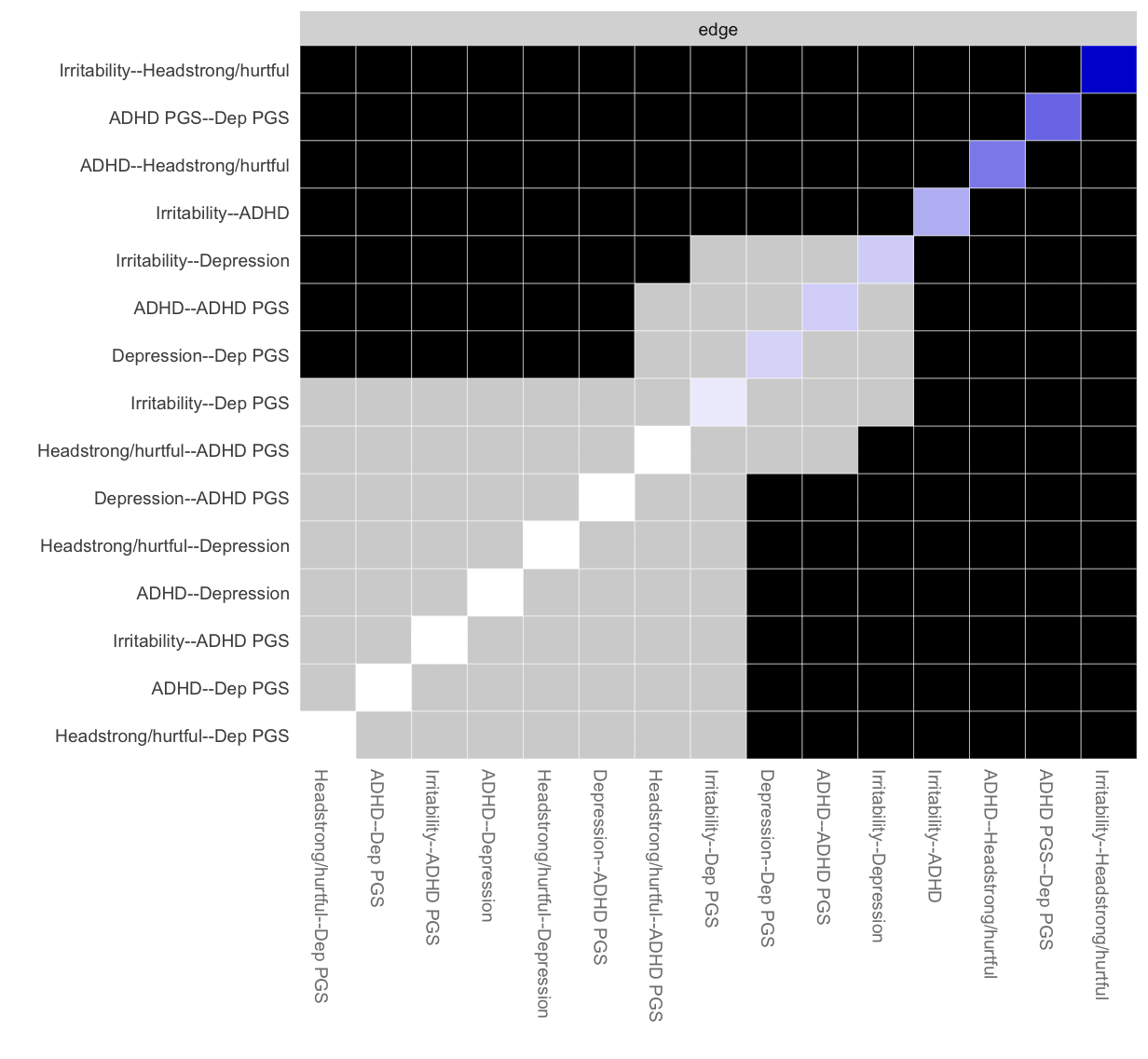


**Figure S1** – Results of *differenceTest*, testing for differences in bootstrapped edge weights, as determined using the presence of overlapping confidence intervals. Black squares indicate edges which significantly differ in weight, grey squares where edges do not.

**Figure S2** – A) ‘Phenotype + PGS’ network containing only ADHD PGS. B) ‘Phenotype + PGS’ network containing only Depression PGS.


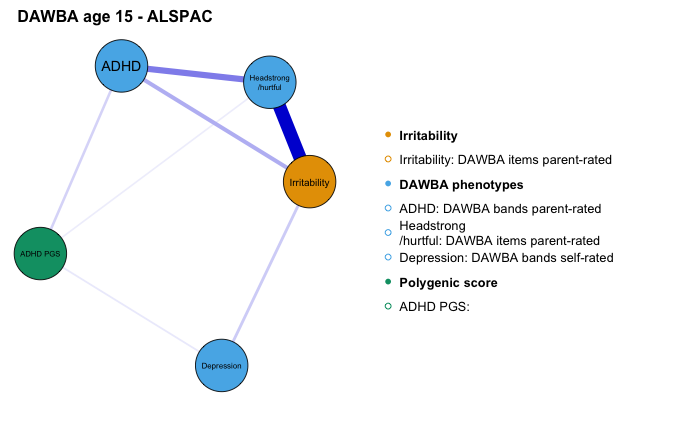

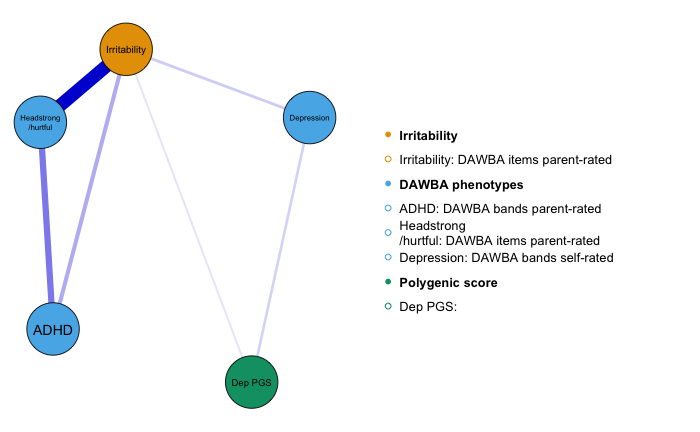


**A**

**B**

**DAWBA age 15 – ALSPAC, individual PGS**


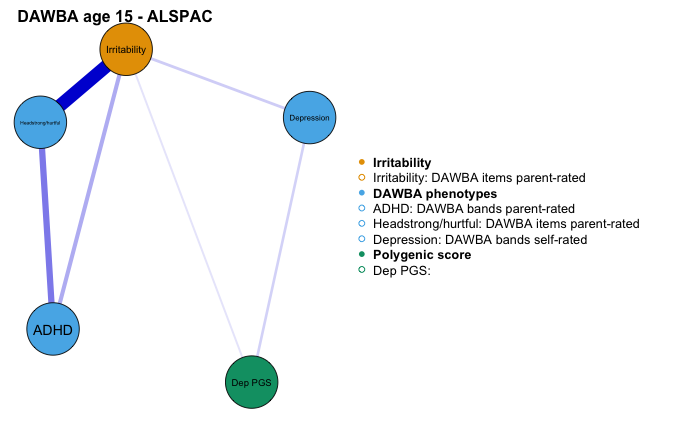

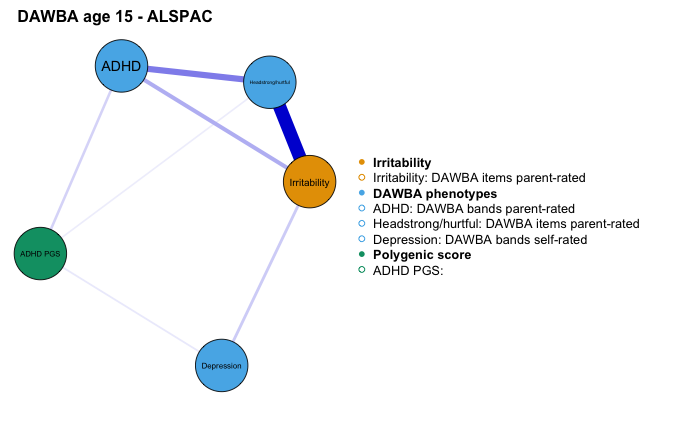


**Figure S3** – A) ‘Phenotype only’ network estimated with complete cases (n=4,529).


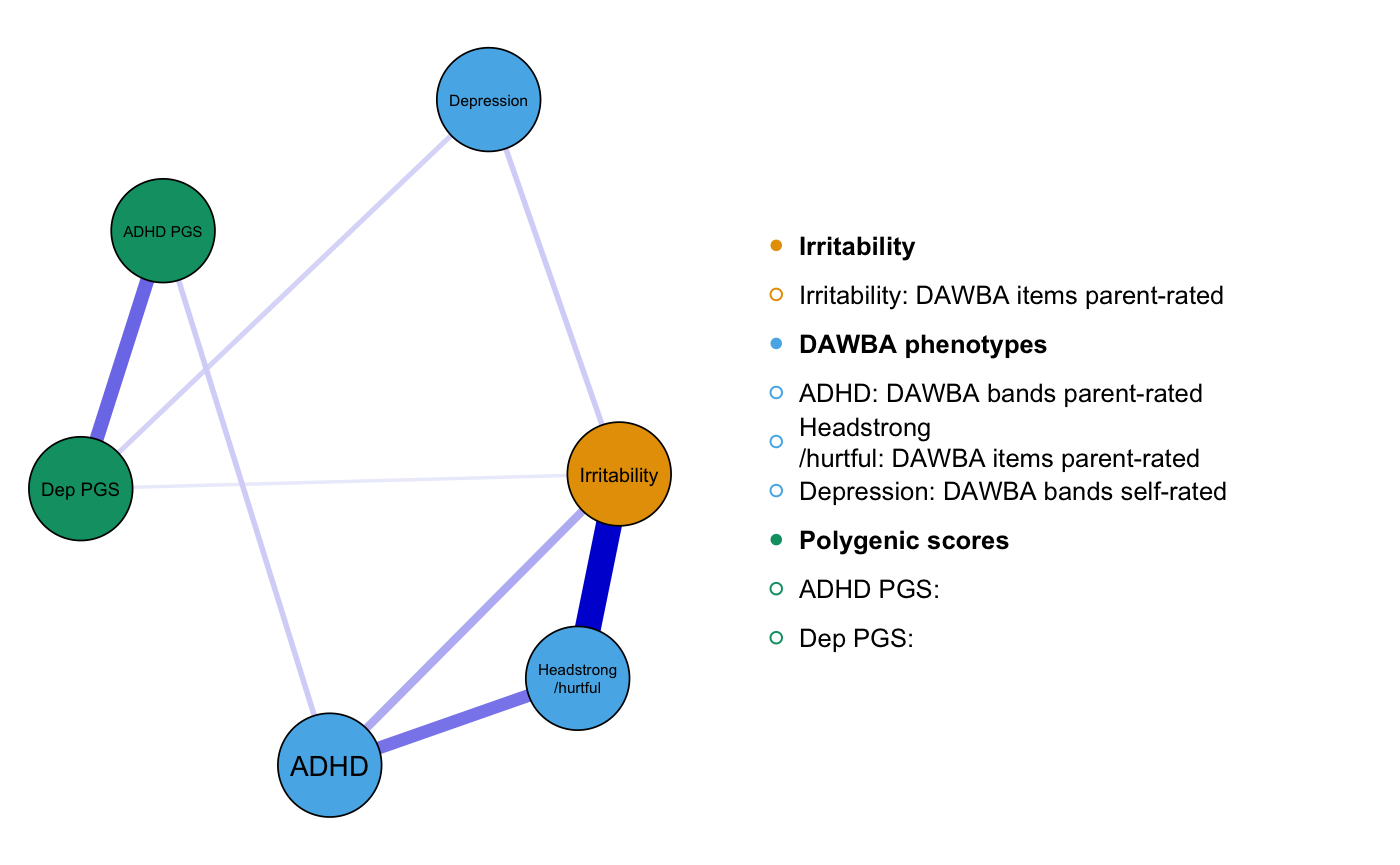

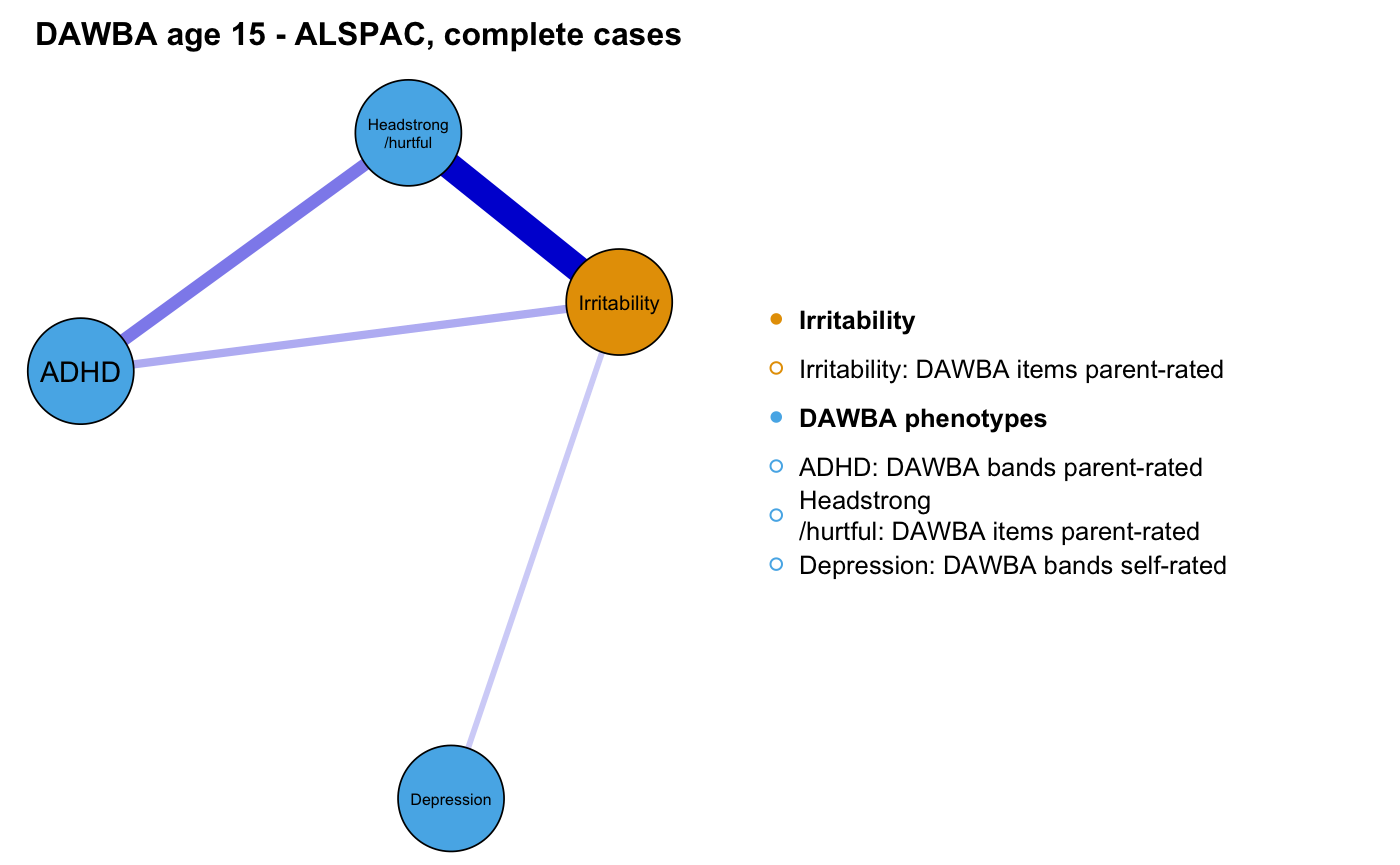


**A**

**B**


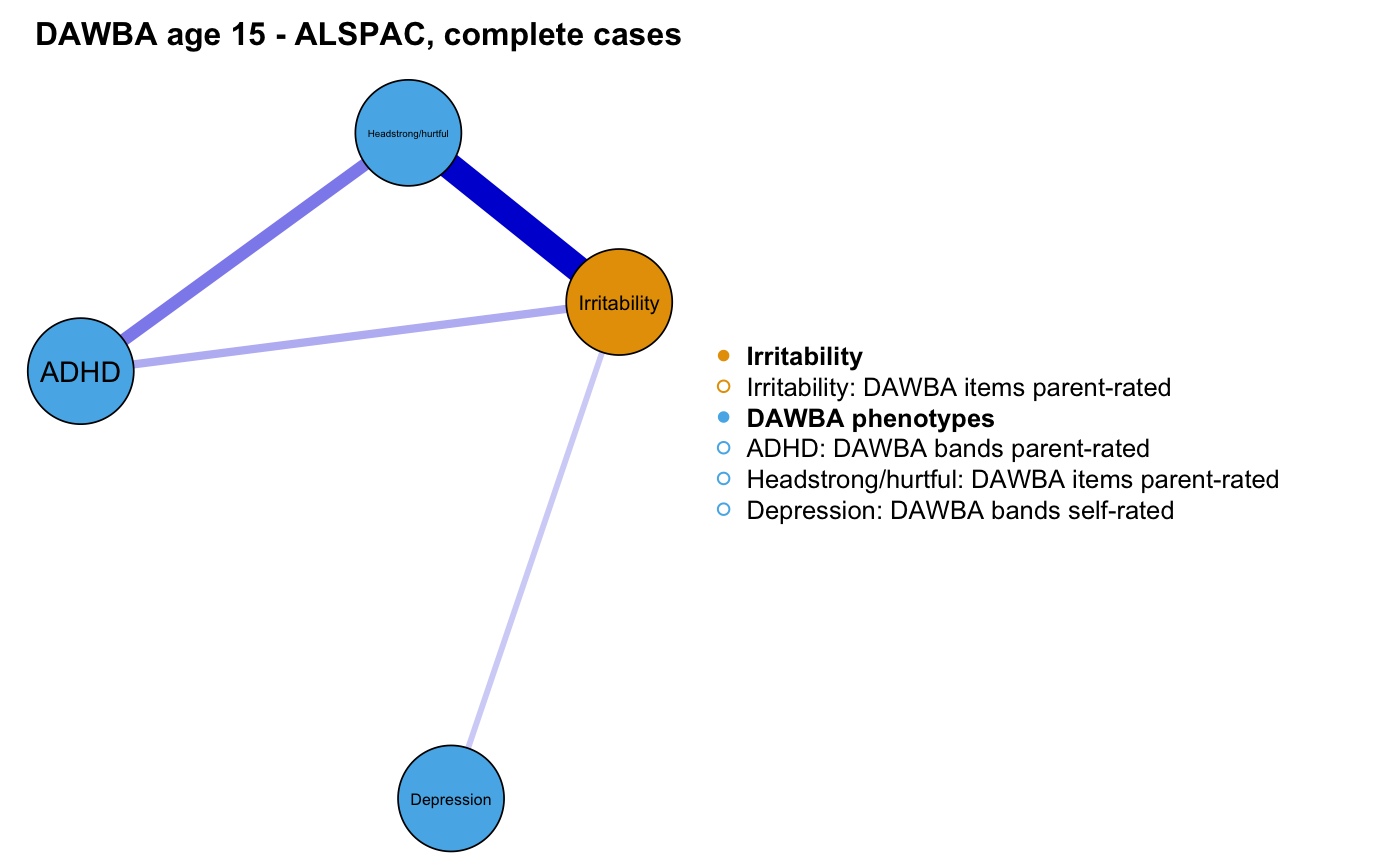

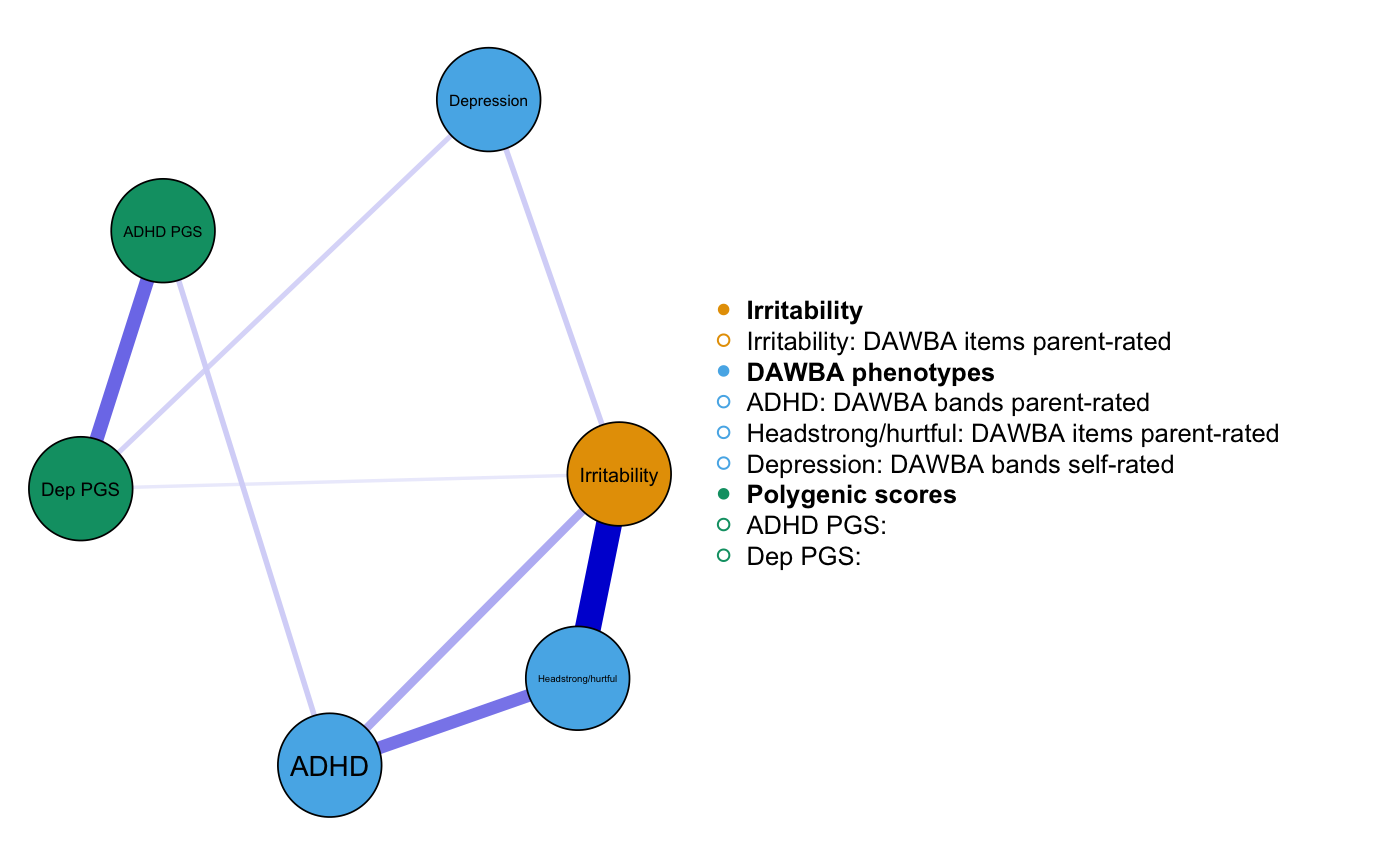


B) ‘Phenotype + PGS’ network estimated with complete cases (n=3,732).


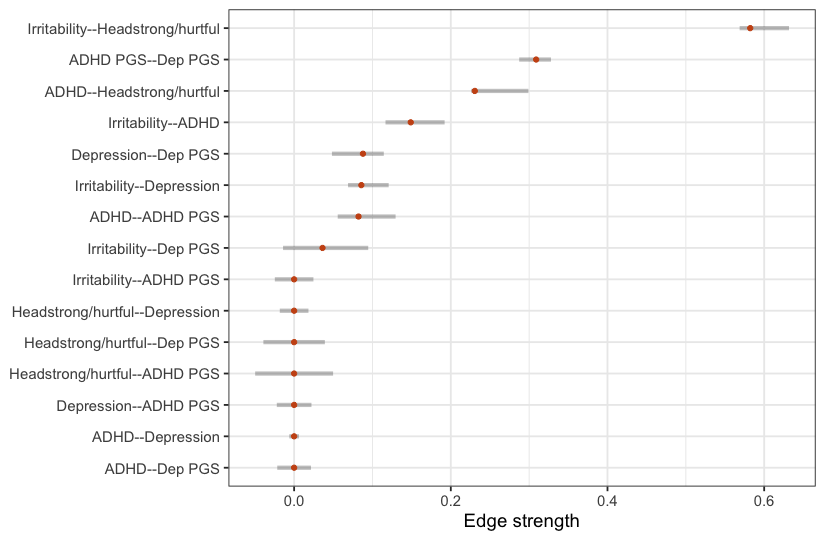


**Figure S4** – Edge strengths (orange dots) of network estimated from imputed datasets, with range bars indicating 95% confidence intervals (CI) of sample network edge strength from bootstraps. All imputed edge strengths lie within CI range.


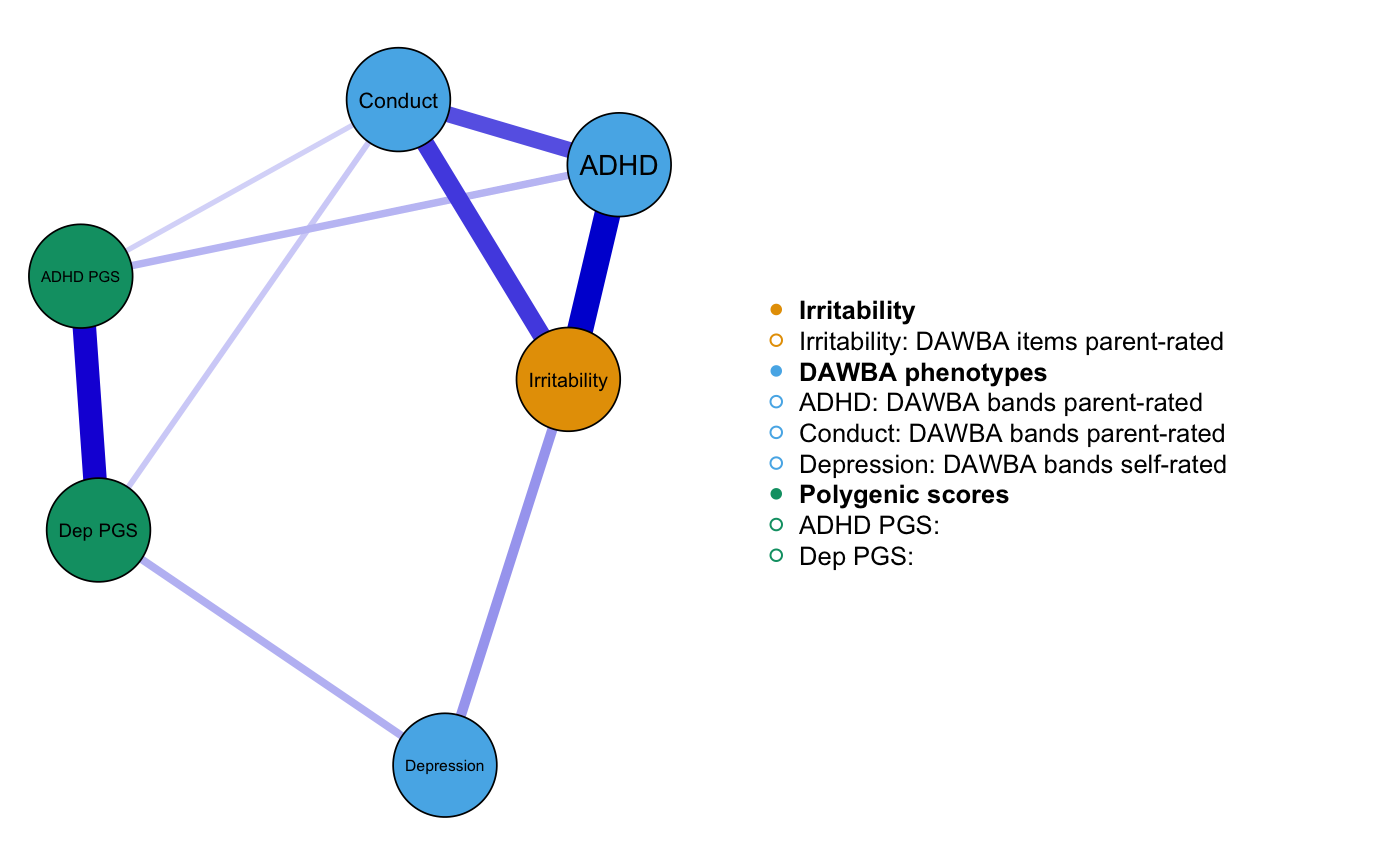

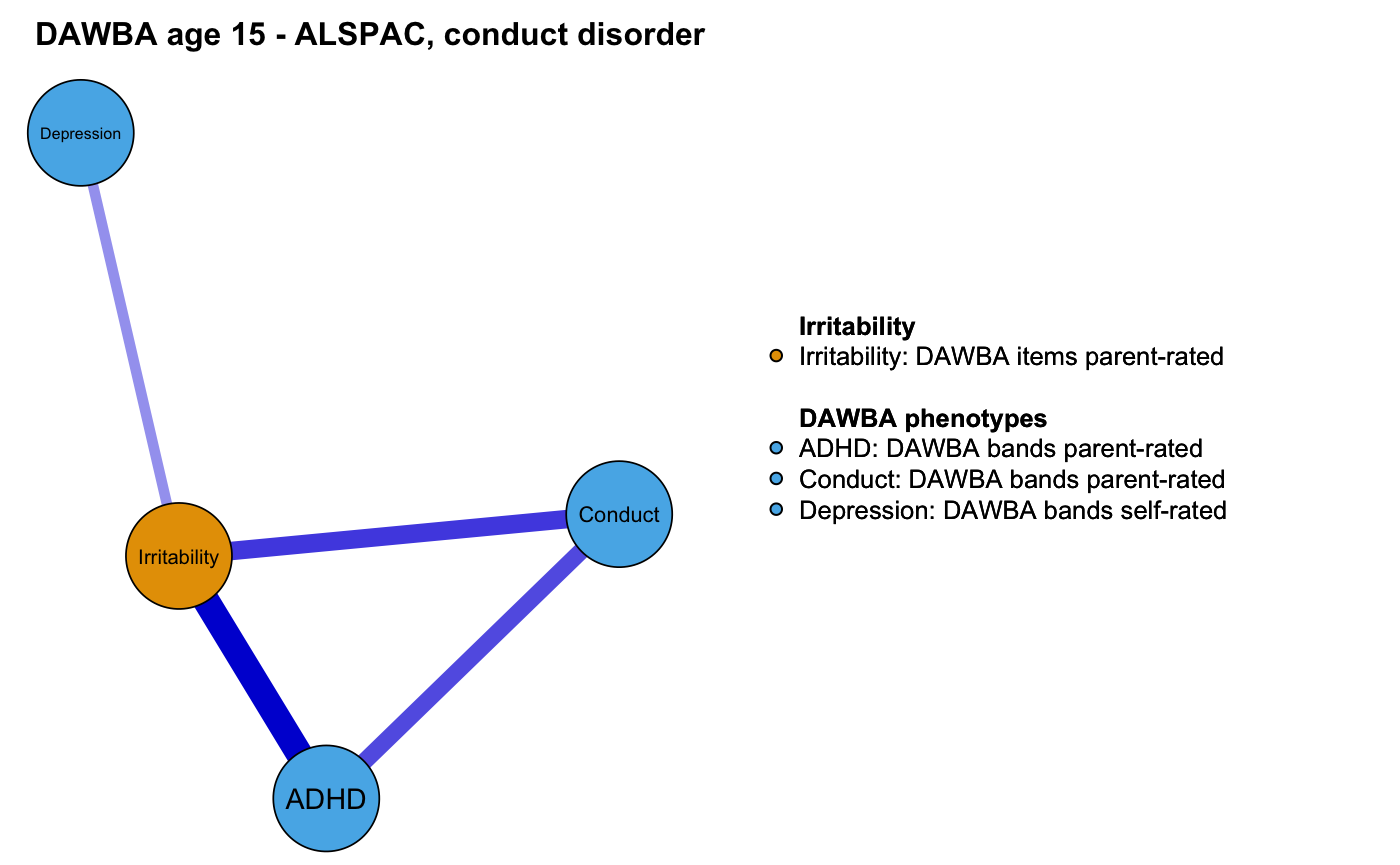


**A)**

**B)**

**Figure S5 –** A) ‘Phenotype only’ network using DAWBA variables at age 15 in ALSPAC, including a node for conduct disorder instead of headstrong/hurtful ODD symptoms. B) ‘Phenotype + PGS’ network using DAWBA variables at age 15 in ALSPAC. In both networks edges represent Spearman’s partial correlations between nodes. Irritability, ADHD and conduct disorder phenotypes are parent-reported; depression phenotype is self-reported.

**
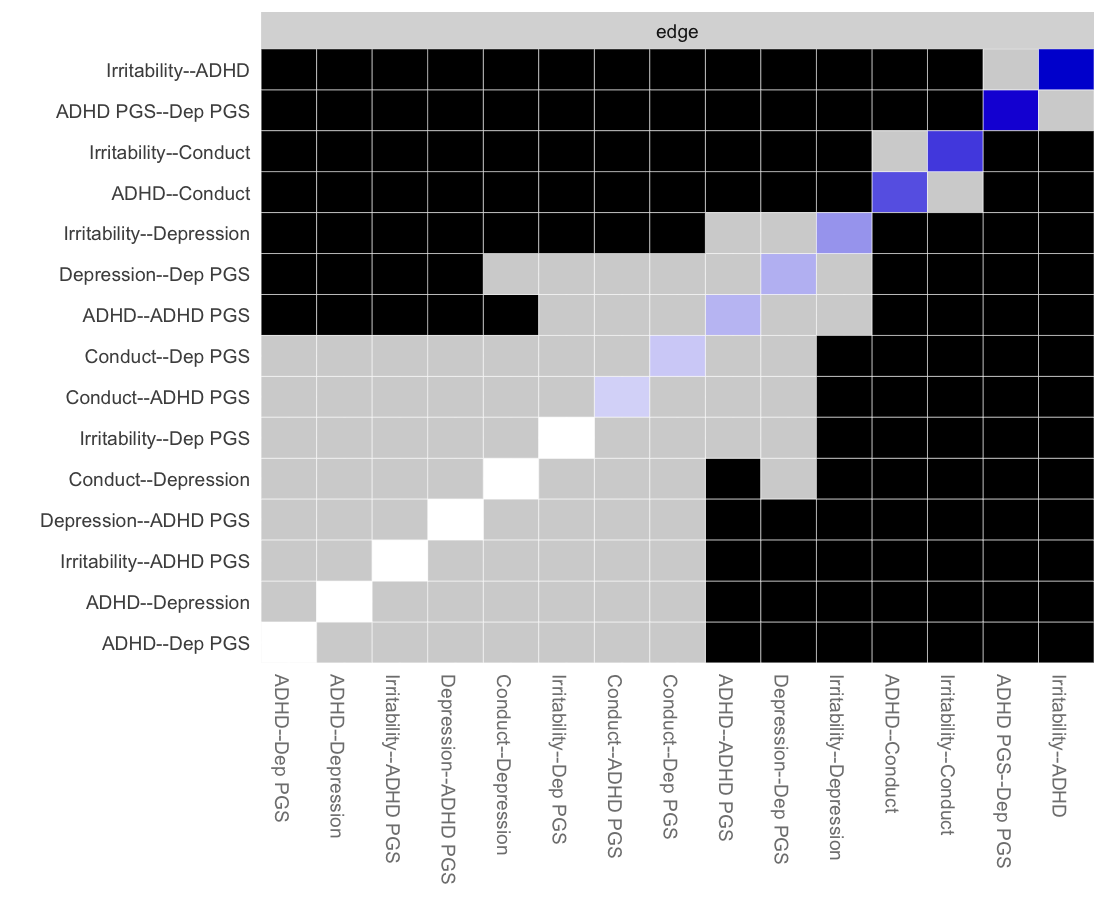
**

**Figure S6 -** Results of *differenceTest* the network including conduct disorder instead of the headstrong/hurtful ODD dimension to capture behavioural problems. This method tests for differences in bootstrapped edge weights, as determined using the presence of overlapping confidence intervals. Black squares indicate edges which significantly differ in weight, grey squares where edges do not.

**Figure S7** – ‘Phenotype + PGS’ network estimated in females (top) and males (bottom).


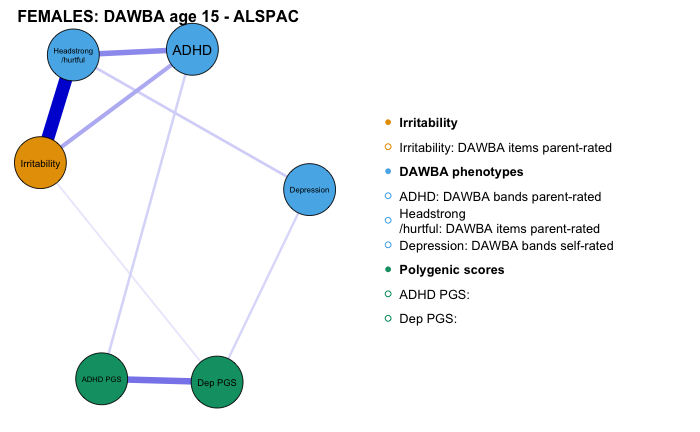

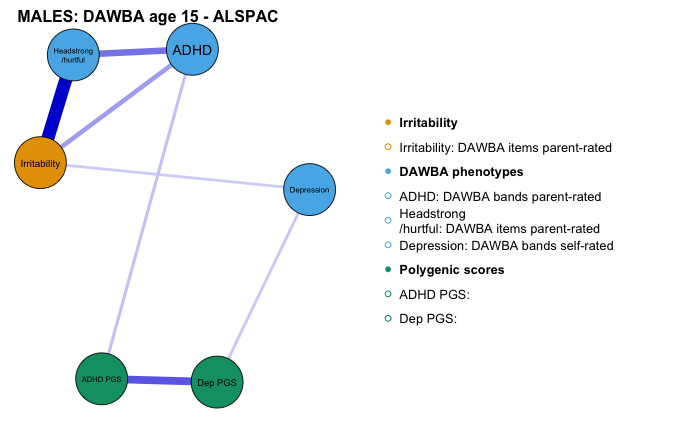

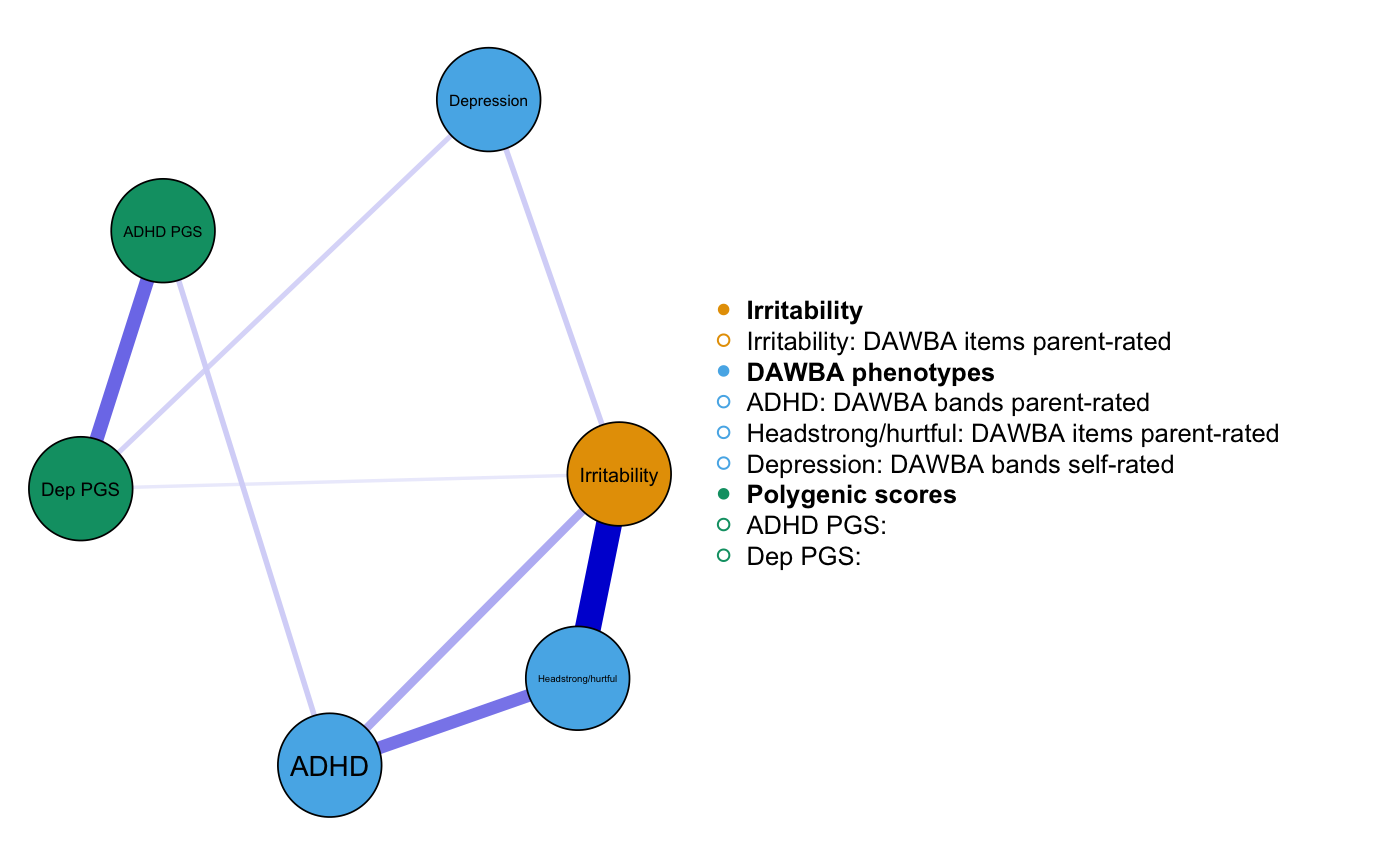


**
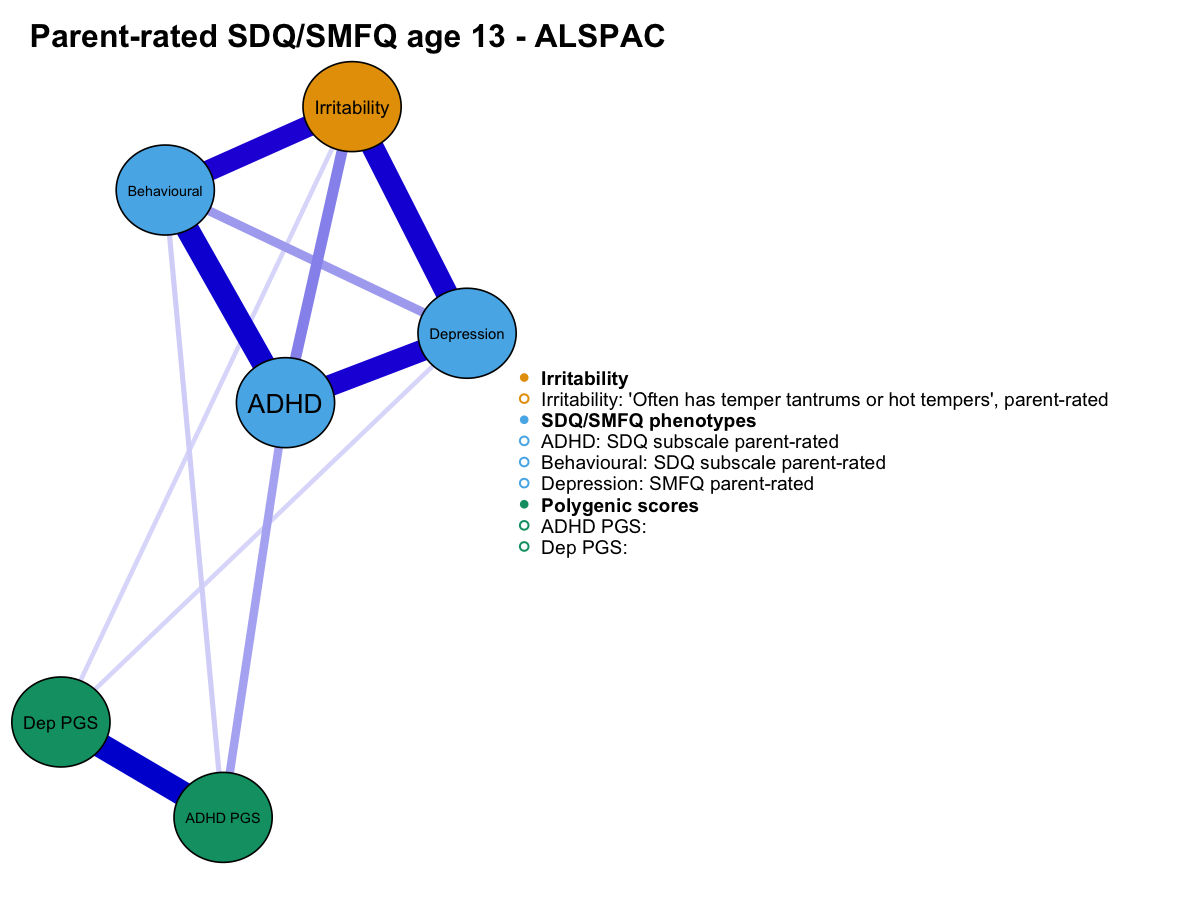
**

**Figure S8** - ‘Phenotype + PGS’ network using all parent-reported SDQ/SMFQ phenotypes.


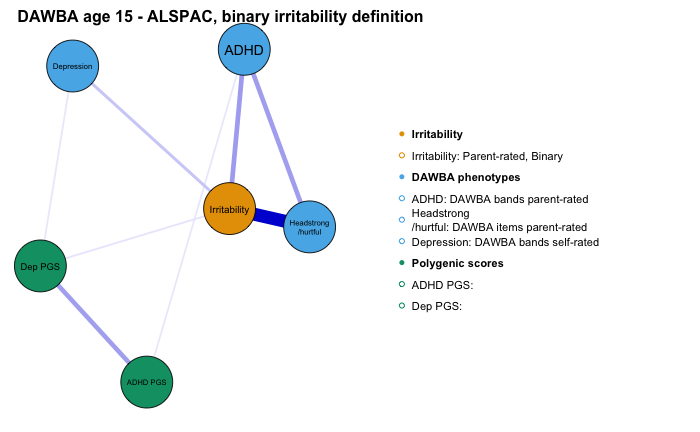

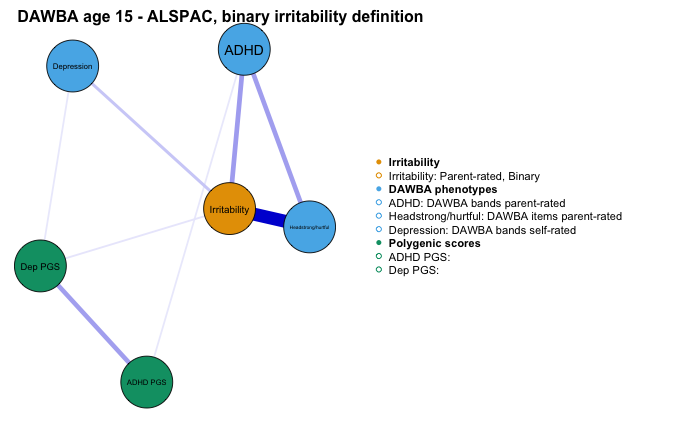


**Figure S9** – Phenotype + PGS network estimated using a binary definition of irritability based on DAWBA in ALSPAC.

**A**

**B**


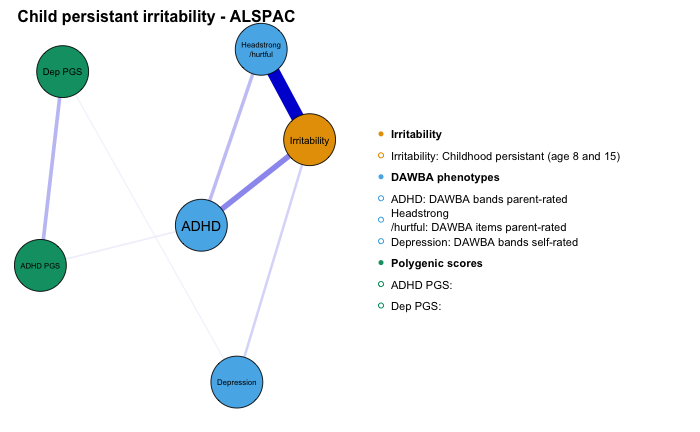

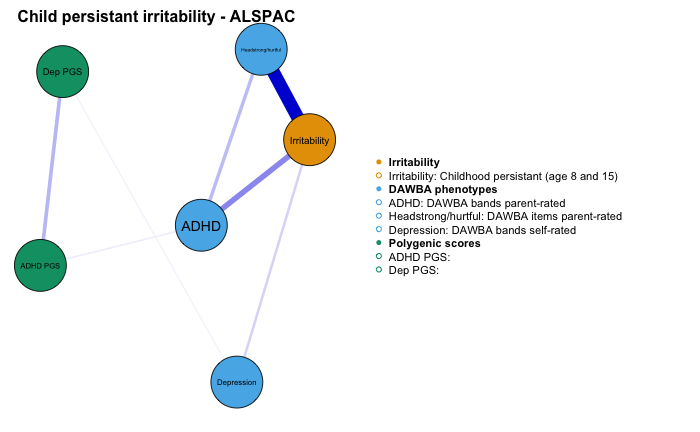

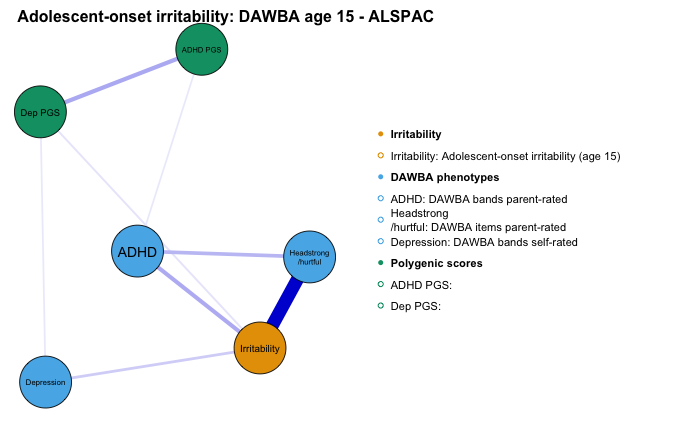

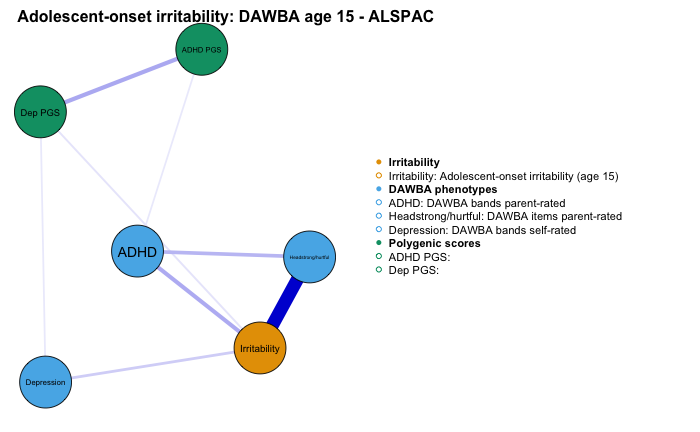


**Figure S10** – Phenotype & PGS networks estimated using irritability definitions based on age of onset. A) Child-persistent irritability and B) adolescent-onset irritability networks.

**References**

Adams, M. J., Streit, F., Meng, X., Awasthi, S., Adey, B. N., Choi, K. W., Chundru, V. K., Coleman, J. R. I., Ferwerda, B., Foo, J. C., Gerring, Z. F., Giannakopoulou, O., Gupta, P., Hall, A. S. M., Harder, A., Howard, D. M., Hübel, C., Kwong, A. S. F., Levey, D. F., Mitchell, B. L., . . . Mcintosh, A. M. (2025). Trans-ancestry genome-wide study of depression identifies 697 associations implicating cell types and pharmacotherapies. *Cell,* 188**,** 640-652.e649.

Demontis, D., Walters, G. B., Athanasiadis, G., Walters, R., Therrien, K., Nielsen, T. T., Farajzadeh, L., Voloudakis, G., Bendl, J., Zeng, B., Zhang, W., Grove, J., Als, T. D., Duan, J., Satterstrom, F. K., Bybjerg-Grauholm, J., Bækved-Hansen, M., Gudmundsson, O. O., Magnusson, S. H., Baldursson, G., . . . I, P.-B. C. (2023). Genome-wide analyses of adhd identify 27 risk loci, refine the genetic architecture and implicate several cognitive domains. *Nature Genetics,* 55**,** 198-208.

Elsworth, B., Lyon, M., Alexander, T., Liu, Y., Matthews, P., Hallett, J., Bates, P., Palmer, T., Haberland, V., Smith, G. D., Zheng, J., Haycock, P., Gaunt, T. R. & Hemani, G. (2020). The mrc ieu opengwas data infrastructure. *bioRxiv***,** 2020.2008.2010.244293.

Epskamp, S., Borsboom, D. & Fried, E. I. (2018). Estimating psychological networks and their accuracy: A tutorial paper. *Behavior Research Methods,* 50**,** 195-212.

Ge, T., Chen, C. Y., Ni, Y., Feng, Y. A. & Smoller, J. W. (2019). Polygenic prediction via bayesian regression and continuous shrinkage priors. *Nat Commun,* 10**,** 1776.

Goodman, R., Ford, T., Richards, H., Gatward, R. & Meltzer, H. (2000). The development and well-being assessment: Description and initial validation of an integrated assessment of child and adolescent psychopathology. *J Child Psychol Psychiatry,* 41**,** 645-655.

Henriksen, M., Skrove, M., Hoftun, G. B., Sund, E. R., Lydersen, S., Kalvin, C. B. & Sukhodolsky, D. G. (2021). Exposure to traumatic events poses greater risk for irritability in girls than in boys. *Journal of Affective Disorders Reports,* 6**,** 100204.

International Hapmap, C., Altshuler, D. M., Gibbs, R. A., Peltonen, L., Altshuler, D. M., Gibbs, R. A., Peltonen, L., Dermitzakis, E., Schaffner, S. F., Yu, F., Peltonen, L., Dermitzakis, E., Bonnen, P. E., Altshuler, D. M., Gibbs, R. A., De Bakker, P. I., Deloukas, P., Gabriel, S. B., Gwilliam, R., Hunt, S., . . . Mcewen, J. E. (2010). Integrating common and rare genetic variation in diverse human populations. *Nature,* 467**,** 52-58.

Isvoranu, A. M. & Epskamp, S. (2023). Which estimation method to choose in network psychometrics? Deriving guidelines for applied researchers. *Psychol Methods,* 28**,** 925-946.

Krebs, G., Bolhuis, K., Heyman, I., Mataix-Cols, D., Turner, C. & Stringaris, A. (2013). Temper outbursts in paediatric obsessive-compulsive disorder and their association with depressed mood and treatment outcome. *Journal of Child Psychology and Psychiatry,* 54**,** 313-322.

Stringaris, A. & Goodman, R. (2009). Three dimensions of oppositionality in youth. *J Child Psychol Psychiatry,* 50**,** 216-223.
